# Myeloid Cell Hypoxia-Inducible Factors Promote Resolution of Inflammation in Experimental Colitis

**DOI:** 10.3389/fimmu.2018.02565

**Published:** 2018-11-05

**Authors:** Nan Lin, Jessica E. S. Shay, Hong Xie, David S. M. Lee, Nicolas Skuli, Qiaosi Tang, Zilu Zhou, Andrew Azzam, Hu Meng, Haichao Wang, Garret A. FitzGerald, M. Celeste Simon

**Affiliations:** ^1^Perelman School of Medicine, Abramson Family Cancer Research Institute, University of Pennsylvania, Philadelphia, PA, United States; ^2^Department of Cancer Biology, Perelman School of Medicine, University of Pennsylvania, Philadelphia, PA, United States; ^3^Genomics and Computational Biology Graduate Program, Perelman School of Medicine, University of Pennsylvania, Philadelphia, PA, United States; ^4^Division of Gastroenterology, Department of Medicine, University of Pennsylvania, Philadelphia, PA, United States; ^5^Abramson Cancer Center, University of Pennsylvania, Philadelphia, PA, United States; ^6^Perelman School of Medicine, Institute for Translational Medicine and Therapeutics, University of Pennsylvania, Philadelphia, PA, United States; ^7^Department of Emergency Medicine, North Shore University Hospital, Manhasset, NY, United States; ^8^The Feinstein Institute for Medical Research, Manhasset, NY, United States; ^9^Department of Cell and Developmental Biology, Perelman School of Medicine, University of Pennsylvania, Philadelphia, PA, United States

**Keywords:** inflammation, colitis, macrophages, neutrophils, hypoxia, HIF, serum amyloid A

## Abstract

Colonic tissues in Inflammatory Bowel Disease (IBD) patients exhibit oxygen deprivation and activation of hypoxia-inducible factor 1α and 2α (HIF-1α and HIF-2α), which mediate cellular adaptation to hypoxic stress. Notably, macrophages and neutrophils accumulate preferentially in hypoxic regions of the inflamed colon, suggesting that myeloid cell functions in colitis are HIF-dependent. By depleting ARNT (the obligate heterodimeric binding partner for both HIFα subunits) in a murine model, we demonstrate here that myeloid HIF signaling promotes the resolution of acute colitis. Specifically, myeloid pan-HIF deficiency exacerbates infiltration of pro-inflammatory neutrophils and Ly6C^+^ monocytic cells into diseased tissue. Myeloid HIF ablation also hinders macrophage functional conversion to a protective, pro-resolving phenotype, and elevates gut serum amyloid A levels during the resolution phase of colitis. Therefore, myeloid cell HIF signaling is required for efficient resolution of inflammatory damage in colitis, implicating serum amyloid A in this process.

## Introduction

Hypoxia (low oxygen tension) is evident in many pathological contexts, including chronic intestinal inflammation, commonly known as inflammatory bowel diseases (IBD) (1, 2). Healthy colon tissue exhibits O_2_ partial pressures ranging from ~85 mmHg in intestinal crypts to < 10 mmHg in villus tips (3). This “physiological” hypoxia is profoundly exacerbated in the context of active inflammation, as revealed in murine models of IBD (1, 2, 4). A well-characterized cellular response to O_2_ deprivation is activation of hypoxia-inducible factor (HIF) transcriptional regulators, comprised of an O_2_-sensitive α-subunit (HIF-1α or HIF-2α) and a constitutively expressed β subunit (HIF-1β, or aryl hydrocarbon receptor nuclear translocator [ARNT]). In the presence of O_2_, HIF-α is hydroxylated by prolyl hydroxylase domain-containing proteins (PHDs), and then poly-ubiquitinated by the von Hippel-Lindau (pVHL) tumor suppressor E3 ubiquitin ligase complex, leading to its degradation by the 26S proteasome (5–7). Hypoxic conditions inhibit PHD enzymes, allowing HIF-α subunits to accumulate, dimerize with their obligate binding partner ARNT, and bind to hypoxia-response elements (HREs) to enhance transcription of hundreds of genes whose products mediate cellular adaptation to hypoxia, including glycolysis, angiogenesis, and inflammatory responses (8–10). As hypoxia is a prominent characteristic of inflamed colon tissue, activation of both HIF-1α and HIF-2α, the two best-characterized HIF-α subunits, is also frequently detected in the colon of IBD patients (11–13).

Inflamed tissues can become hypoxic due to abnormal vascular function (14) and enhanced metabolic activities of bacteria and infiltrating immune cells, such as myeloid cells, which include granulocytes (i.e., neutrophils, basophils, eosinophils and mast cells) and monocytes that differentiate into macrophages and dendritic cells (10, 15). When recruited to sites of inflammation, these cells eliminate invading pathogens by driving innate immune responses, e.g., phagocytosis or inflammatory cytokine secretion. Macrophages and neutrophils accumulate in the mucosa of IBD patients (16–20) and play critical roles in modulating and resolving inflammation (21–27). Moreover, their preferential localization within hypoxic regions suggests a potential role of O_2_ limitation in dictating myeloid cell inflammatory responses. Significant effort has elucidated that HIF-1α and HIF-2α in myeloid cells (10) have common (28–31), non-redundant (28, 29), and even opposing functions (32, 33), reflecting the complexity of HIF function in these cells.

ARNT depletion represents an attractive approach to study pan-HIF inhibition in multiple contexts (34–36). In a murine model of colitis, pharmacological HIF stabilization using PHD inhibitors proved to be protective, partly through anti-apoptotic effects on epithelial cells (37–39). Notably, HIF-1α and HIF-2α can oppose each other during intestinal inflammation: for example, epithelial cell HIF-1α helps maintain intestinal barrier functions during colitis (4, 40, 41), whereas HIF-2α worsens colitis by promoting tumor necrosis factor alpha (TNFα) production in epithelial cells (12). HIF functions can also differ drastically depending on cell type. For example, dendritic cell-specific HIF-1α suppresses intestinal inflammation via activation of regulatory T cells (42), whereas macrophage-specific HIF-1α has been implicated as pathogenic (43, 44). However, the role(s) of pan-HIF activation specifically in myeloid cells during colitis are yet to be fully investigated.

In this study, we show that myeloid HIF-α/ARNT heterodimers are required for efficient resolution of inflammation in a dextran sulfate sodium (DSS)-induced acute colitis murine model, and confirm that these effects are due to disruption of HIF-1α and HIF-2α signaling. Lamina propria neutrophil and monocyte numbers are elevated in mice with myeloid HIF deficiency during the resolution phase of acute colitis. Microarray analysis of colonic macrophages indicates that their conversion to a “pro-resolving” profile requires a full complement of HIF activities. We also identify serum amyloid As (SAAs) as a likely mechanism through which HIF-deficient macrophages contributes to aberrant disease resolution. Our findings are the first to connect HIFs to SAAs in colitis, and highlight potential clinical benefits of activating myeloid HIF signaling as a way to resolve intestinal inflammation.

## Methods

### Mice

The *Arnt, Hif1*α, and *Hif2*α conditional alleles were crossed with the LysM-Cre allele (45) to achieve myeloid-specific *Arnt, Hif1*α, or *Hif2*α conditional knockout mice. Mice with *Hif1a* conditional allele on a C57BL/6 background were purchased from Jackson Laboratory (Bar Harbor, ME). *LysMCre;Hif2*α^*fl*/*fl*^ mice were generated and described in a previous study (29). Ever since this study, we have backcrossed *Hif2*α^*fl*/*fl*^ mice with C57BL/6 mice sufficiently to ensure a similar background to other strains. *Arnt*^*fl*/*fl*^ *mice* with a mixed background of C57BL/6 and 129svJ were also backcrossed with C57BL/6 mice sufficiently before crossed with *LysMCre* mice. *In vivo* experiments using *LysMCre* and *LysMCre;Arnt*^*fl*/*fl*^ mice were carried out using 24 mice in each cohort. The experiments with either *LysMCre;Hif1*α^*fl*/*fl*^ or *LysMCre;Hif2*α^*fl*/*fl*^ were performed with relatively small numbers of mice (*n* = 4) for experimental and control groups as a confirmation that phenotypes observed with *LysMCre;Arnt*^*fl*/*fl*^ mice (*n* = 24) are HIF dependent. All animal procedures were performed in accordance with NIH guidelines and were approved by the Institutional Animal Care and Use Committee of the University of Pennsylvania.

### Induction of colitis and clinical scoring

Dextran sulfate sodium (DSS) (MW 36–50 kDa, MP Biomedicals, Santa Ana, CA) was administered orally in drinking water at 3% (w/v) concentration for 5 days followed by normal drinking water for 3 days. Mice of both genotypes were housed in the same cages to minimize potential confounding influences from differing microbiomes. Body weight, stool consistency, and fecal blood were monitored and recorded daily for each mouse. Disease Activity Index (DAI) was calculated as the sum of scores for body weight loss, stool consistency, and fecal blood. These three parameters were scored as following (46, 47): 0, no weight loss or < 1% weight loss, normal stool pellets, negative Hemoccult test (Beckman Coulter, Brea, CA); 1, 1–5% weight loss, slightly loose feces; 2, 5–10% weight loss, lose feces, positive Hemoccult test; 3, 10–20% weight loss, watery diarrhea; 4, more than 20% weight loss, positive Hemoccult test, and visible fecal and rectal blood.

### Histopathology assessment of DSS-induced colitis

Colons ranging from cecum to rectum were cut longitudinally, fixed in 4% paraformaldehyde/PBS (4°C overnight), and embedded in paraffin for sectioning. Five-μm thick sections were cut and stained with hematoxylin and eosin and scored in a double-blind manner. Tissue sections were scored for loss of mucosal architecture, cellular infiltration, crypt abscess formation, Goblet cell depletion, and tissue affected, yielding a total histopathology score. Loss of mucosal architecture was scored 0 to 3 for absent, mild, moderate, and severe with loss of entire crypts. Cellular infiltration was scored 0 to 3 for absent, mild, moderate, and extensive. Crypt abscess formation was scored 0 or 1 for absent or present. Goblet cell depletion was scored 0 or 1 for absent or present. Percentage of tissue affected was scored 0 to 3 for absent, >10, 20–30, and 40–50%. The sum of these values for each mouse gave a total histopathology score.

### Isolation of lamina propria cells

Lamina propria cells were isolated using a modified version of previously described protocols (48, 49). Briefly, colons were cut open longitudinally and shaken in medium with 1 mM EDTA and 1 mM DTT twice for 20 min each at 37°C. The remaining tissue was further digested with 0.5 mg/mL Collagenase/Dispase (Roche, Basel, Switzerland) and 0.05 mg/mL (92.15 Kunitz unit/mL) DNase I (Sigma-Aldrich, St. Louis, MO) for 40 min at 37°C with agitation. Cells were then harvested by passing the suspension through a 70-μm cell strainer (Corning, Corning, NY). Single cell suspensions were later analyzed *ex vivo* by flow cytometry.

### Flow cytometry

Single cells suspensions were blocked with Mouse BD Fc Block™ (BD Biosciences, Franklin Lakes, NJ) for 10 min and then stained in FACS buffer (PBS with 4% FBS and 2 mM EDTA) with the following fluorochrome-conjugated antibodies: APC-conjugated anti-CD19 (1D3, #550992, 1:200), APC-Cy7-conjugated anti-CD4 (GK1.5, #552051, 1:200), PE-Cy7-conjugated anti-CD8a (53-6.7, #552877, 1:200), FITC-conjugated anti-CD45 (30-F11, #561088, 1:100), V450-conjugated anti-CD3e (500A2, 560801, 1:100), APC-Cy7-conjugated anti-Ly6C (AL-21, #560596, 1:100), PE-Cy7-conjugated anti-CD45 (30-F11, #552848, 1:100), V450-conjugated anti-CD11c (HL3, #560521, 1:100), PerCP-Cy5.5-conjugated anti-CD11b (M1/70, #561114, 1:100), AF700-conjugated anti-Ly6G (1A8, #561236, 1:100) (from BD Biosciences); PE-conjugated anti-F4/80 (BM8, #12-4801, 1:100), AF700-conjugated anti-CD25 (PC61.5, #56-0251-80, 1:100) (from eBioscience, San Diego, CA). Viability was determined by staining cells with LIVE/DEAD™ Fixable Aqua Dead Cell stain, 1:300 (Thermo Fisher Scientific, Waltham, MA). Flow cytometry was performed on a LSR A flow cytometer (BD Biosciences), and data were analyzed using FlowJo software.

### Colonic explant supernatant collection and ELISA

A 0.5 cm-long colon segment was collected about 1 cm from the rectum from each well-flushed mouse colon. These colon segments were cultured in 24-well plates containing 0.6 mL of complete tissue culture medium (DMEM with 25 mM HEPES, 0.05 mM 2-mercaptoethanol, 2 mM L-Glutamine, 100 U/mL Penicillin, 100 μg/mL Streptomycin, and 10% FBS) for 24 h till cell-free culture supernatant was collected. The collected supernatants were then subject to quantification of cytokine levels using the following ELISA kits: Mouse IL-1 beta/IL-1F2 DuoSet ELISA kit (#DY401-05), Mouse IL-6 DuoSet ELISA (#DY406-05), Mouse IL-10 DuoSet ELISA (#DY417-05), Mouse CXCL1/KC DuoSet ELISA (#DY453-05), Mouse Serum Amyloid A DuoSet ELISA (#DY2948-05), Mouse Cytokine Antibody Array, Panel A (#ARY006) (from R&D Systems, Minneapolis, MN), and Mouse SAA-3 ELISA (#EZMSAA3-12K) (from Millipore Sigma, Burlington, MA).

### Microarray analysis

Total RNA was extracted from flow cytometry-sorted lamina propria macrophages from *LysMCre* and *LysMCre;Arnt*^*fl*/*fl*^ mice using TRIzol™ LS Reagent (Thermo Fisher Scientific). Total RNA quality control tests were determined using BioAnalyzer 2100 (Agilent) and Nanodrop spectrophotometry (Thermo Fisher Scientific). The cDNA was prepared, labeled, and hybridized to Affymetrix GeneChip, mouse gene 2.0 using GeneChip WT PLUS Reagent Kit (Affymetrix, Santa Clara, CA). Hybridized chips were scanned with GeneChip™ Scanner 3000 7G (Affymetrix). Affymetrix Command Console and Expression Console (Thermo Fisher Scientific) were used to quantitate expression levels for targeted genes; default values provided by Affymetrix were applied to all analysis parameters. Transcriptomic analysis was carried out using Partek Genomic Suite 6.1 (Partek, Inc., St. Louis, MO). Robust MultiArray Average (RMA) (50) was used for normalization of the raw probe intensity data. Significance Analysis of Microarrays (SAM) (51) was applied to compare *LysMCre* and *LysMCre;Arnt*^*fl*/*fl*^ samples. The magnitude of d score, the *T*-statistic value used in SAM, scales with statistical significance. A false discovery rate (q-value) was estimated by SAM based on a null distribution for the d score by permuting the samples with respect to *LysMCre* and *LysMCre;Arnt*^*fl*/*fl*^ classes. Differentially expressed genes were defined as those having fold change above or below 1.5 and *q*-value < 0.05. All of our microarray data have been deposited in the Gene Expression Omnibus (http://www.ncbi.nlm.nih.gov/geo) with the accession number [GSE121078].

### Generation and culture of bone marrow-derived macrophages (BMDMs) and neutrophils (BMDNs)

Bone marrow cells were isolated from femurs and tibias of *LysMCre* and *LysMCre;Arnt*^*fl*/*fl*^ mice. After a quick incubation in ammonium-chloride-potassium (ACK) lysing buffer (Lonza, Basel, Switzerland) to remove red blood cells, the remaining bone marrow cells were cultured in BMDM medium (DMEM with 20% heat-inactivated Hyclone FBS, 30% L929 conditioned medium, 1X Antibiotic-Antimycotic, 2 mM L-Glutamine, and 0.055 mM 2-mercaptoethanol) for 5 days on non-treated tissue culture plates before passaging. To obtain BMDNs, EasySep™ Mouse Neutrophil Enrichment Kit (STEMCELL Technologies, Vancouver, Canada) was used for negative selection of neutrophils from bone marrow cells after lysis by ACK lysing buffer. BMDNs were analyzed immediately or cultured in neutrophil culture medium (RPMI 1640 with 10% FBS, 100 U/mL Penicillin and 100 μg/mL Streptomycin) for up to 24 h before analysis.

### Neutrophil viability/apoptosis assessments

Right after isolation of neutrophils from bone marrow cells using EasySep™ Mouse Neutrophil Enrichment Kit (STEMCELL Technologies), total number of viable cells was determined by cell counting with Trypan Blue. Same number of viable neutrophils was then cultured in neutrophil culture medium (RPMI 1640 with 10% FBS, 100 U/mL Penicillin and 100 μg/mL Streptomycin) for 24 h under normoxia (21% O_2_) or hypoxia (0.5% O_2_) before another viability analysis. Percentage viability was taken as percentage of viable neutrophils after 24-h culture out of viable neutrophils seeded. Caspase-Glo® 3/7 assay (Promega, Madison, WI) was used with these cells at these two time points to assess apoptosis.

### Efferocytosis assay

This was carried out using an efferocytosis assay from Cayman Chemical (Cat #: 601770, Cayman Chemical, Ann Arbor, MI) following a protocol provided by the manufacturers. Briefly, BMDNs and BMDMs were isolated as described in “*Generation and culture of bone marrow-derived macrophages (BMDMs) and neutrophils (BMDNs)*.” BMDNs from *LysMCre* mice were stained with carboxyfluorescein succinimidyl ester (CFSE) for 30 min, and then apoptosis was induced by Staurosporine for 6 h. BMDMs were stained with CytoTell™ Blue and mixed with apoptotic neutrophils at a 3:1 Neutrophil: Macrophage ratio. Cells were then cultured overnight under normoxia or hypoxia (~14 h) before flow cytometry analysis. Macrophages were first gated as CytoTell™ Blue-positive cells, and then macrophages that were positive for CFSE were gated next.

### Quantitative RT-PCR

Total RNA was isolated from colon tissues, macrophages derived from colon, BMDMs, and BMDNs using the RNeasy Mini Kit (Qiagen, Hilden, Germany). For colon tissues, BMDMs, and BMDNs, cDNA was synthesized using a High-Capacity RNA-to-cDNA Master Mix (Applied Biosystems, Foster City, CA). PCR reactions were performed using Taqman Universal PCR reagents mixed with indicated cDNAs and Taqman primers in a ViiA7 Real-Time PCR system (Applied Biosystems). Expression levels were normalized to *Hprt* (Mm01318743_m1). The following Taqman primers were used in this study: *Arnt* (Mm00507836_m1), *Adm* (Mm00437438_g1), *Vegfa* (Mm01281449_m1), *Ldha* (Mm01612132_g1), *Pgk1* (Mm00435617_m1), *Arg1* (Mm00475988_m1), *Serpine1* (MM00435860_M1), *Il1b* (Mm00434228_m1), *Il6* (Mm00446190_m1), *Il12a* (Mm00434169_m1), *Tnf* (Mm00443258_m1), *Cxcl10* (Mm00445235_m1), *Cxcl12* (Mm00445553_m1), *Cxcl13* (Mm04214185_s1), *Ccl4* (Mm00443111_m1), *Ccl5* (Mm01302427_m1), *Cyp1a1* (MM00487218_M1), *Ugt1a1* (Mm02603337_m1), *Cxcr2* (MM99999117_S1), *Il23a* (Mm01160011_g1), *Chi3l3* (Mm00657889_mH), *Mrc1* (Mm00485148_m1), and *Retnla* (Mm00445109_m1). For macrophages isolated from colon, due to limited amount of mRNA, anti-sense RNA (cRNA) generated in preparation for microarray analysis using GeneChip WT PLUS Reagent Kit (Affymetrix) was used to generate cDNA. PCR reactions were performed in a ViiA7 Real-Time PCR system using Sybr Green PCR Master Mix (Invitrogen, Carlsbad, CA) with following primers: mouse *Alox15* (Forward 5′ to 3′: GGCTCCAACAACGAGGTCTAC; Reverse 5′ to 3′: AGGTATTCTGACACATCCACCTT), mouse *Saa1* (Forward 5′ to 3′: ACACCAGGATGAAGCTACTCACCA; Reverse 5′ to 3′: CCCTTGGAAAGCCTCGTGAACAAA), mouse *Saa2* (Forward 5′ to 3″: TGGCTGGAAAGATGGAGACAA; Reverse 5′ to 3′: AAAGCTCTCTCTTGCATCACTG), mouse *Saa3* (Forward 5′ to 3″: TGCCATCATTCTTTGCATCTTGA; Reverse 5′ to 3″: CCGTGAACTTCTGAACAGCCT), mouse *Ptges1* (Forward 5′ to 3′: GGATGCGCTGAAACGTGGA; Reverse 5′ to 3′: CAGGAATGAGTACACGAAGCC), mouse *Ptges2* (Forward 5′ to 3′: AAGGCCATGAATGACCAGGG; Reverse 5′ to 3′: TGTTCGGTACACGTTGGGAG), and mouse *Ptgs2* (Forward 5′ to 3′: TTCAACACACTCTATCACTGGC; Reverse 5′ to 3′: AGAAGCGTTTGCGGTACTCAT).

### Western blot analysis

BMDMs were lysed with RIPA buffer containing protease inhibitor (Thermo Fisher Scientific). Cells lysates were boiled in SDS sample buffer for 10 min, separated by SDS-PAGE, transferred to nitrocellulose membranes, probed with primary antibodies overnight at 4°C, and then detected with horseradish peroxidase-conjugated secondary antibodies (Vector Laboratories, Burlingame, CA) followed by exposure to ECL (PerkinElmer, Waltham, MA). The following antibodies were used at indicated concentration: rabbit anti-ARNT (#5537, 1:1,000, Cell Signaling Technology, Danvers, MA), rabbit anti-HIF-1α (#10006421, 1:1,000, Cayman Chemical), rabbit anti-HIF-2α (#PA1-16510, 1:1,000, Thermo Fisher Scientific), and mouse anti-β-actin (#SC-47778, 1:4,000, Santa Cruz Biotechnology, Dallas, TX).

### Eicosanoids analyses by liquid chromatographymass spectrometry (LC/MS)

Deuterated analogs of Prostaglandin E2-d4 ([d4]-PGE2); Prostaglandin D2-d4 ([d4]-PGD2); Prostaglandin F2α-d4 ([d4]-PGF2α); 6-κ-Prostaglandin F1α-d4 (the hydrolysis product of PGI2) ([d4]-6-κ-PGF1α); Thromboxane B2-d4 (the hydrolysis product of TxA2) ([d4]-TxB2); Leukotriene E4-d5 ([d5]-LTE4); Leukotriene B4-d4 ([d4]-LTB4); 5-Hydroxyeicosatetraenoic acids-d8 ([d8]-5-HETE); 12-Hydroxyeicosatetraenoic acids-d8 ([d8]-12-HETE); 15-Hydroxyeicosatetraenoic acids-d8 ([d8]-15-HETE); Arachidonic Acid-d8 ([d8]-AA); Lipoxin A4-d5 ([d5]-LxA4) and undeuterated resolvin D1, resolvin D2, resolvin E1, Protectin D1 (PD1), and Maresin 1 were purchased from Cayman Chemical.

The analysis of eicosanoid metabolites in colonic explant supernatants and BMDMs/BMDNs culture media was performed as described previously for human plasma (52) with a few modifications. Culture medium samples (450 μl) or colonic supernatant (100 μl) were spiked with stable isotope-labeled internal standards ([d4]-PGE2 [5 ng]; [d4]-PGF2α [2.5 ng]; [d4]-TxB2 [10 ng]; [d4]-LTB4 [1 ng]; [d5]-LTE4 [2.5 ng]; [d8]-5-HETE [2.5 ng]; [d8]-12-HETE [25 ng]; [d8]-15-HETE [1 ng]; [d8]-AA [2,500 ng]; [d_5_]-LxA_4_ [1 ng]) in acetonitrile. The composition of final solutions should be acetonitrile: water = 4:1. Phospholipid and proteins were removed using Phree cartridges. (#8B-S133-TAK from Phenomenex, Torrance, CA). Samples were then dried under a gentle stream of nitrogen at the ambient temperature and reconstituted with 30 μl of methanol. Before injection, 30 μl of water was added to each sample and an aliquot of 20 μl was injected into a C18 UPLC column (ACQUITY UPLC BEH 2.1 × 150 mm × 1.7 μm) (Waters Corporation, Milford, MA) and eluted at a flow rate of 350 μl/min, with a linear gradient from 20% solvent B to 90% in 20 min. Mobile phase A consisted of water/mobile phase B, 95:5 (v/v), with 0.5% formic acid; mobile phase B consisted of acetonitrile/methanol, 95:5 (v/v), with 0.5% formic acid. Mass spectrometer parameters were optimized to obtain maximum sensitivity for respective product ions as described previously (52). The analysis was performed on a Waters ACQUITY Ultra Performance Liquid Chromatography system in-line with a Waters Xevo TQ-S Triple Quadrupole Mass Spectrometer (Waters Corporation). The UPLC system directly interfaced with the negative-mode electrospray ionization (ESI) source of the mass spectrometer using multiple reaction monitoring (MRM). Quantitation was done by peak area ratio and results were normalized to the sample volume.

### Statistical analysis

Data were analyzed with GraphPad Prism 7. Unparied, two tailed *t*-test was used for all single comparisons, and two-way ANOVA followed by Bonferroni's correction was used for multiple comparisons. Data are presented as mean ± standard error of the mean (S.E.M.); values of *p* < 0.05 were considered statistically significant.

## Results

### ARNT depletion in myeloid cells disrupts both HIF-1α and HIF-2α transcriptional activity

**Figure 1:**
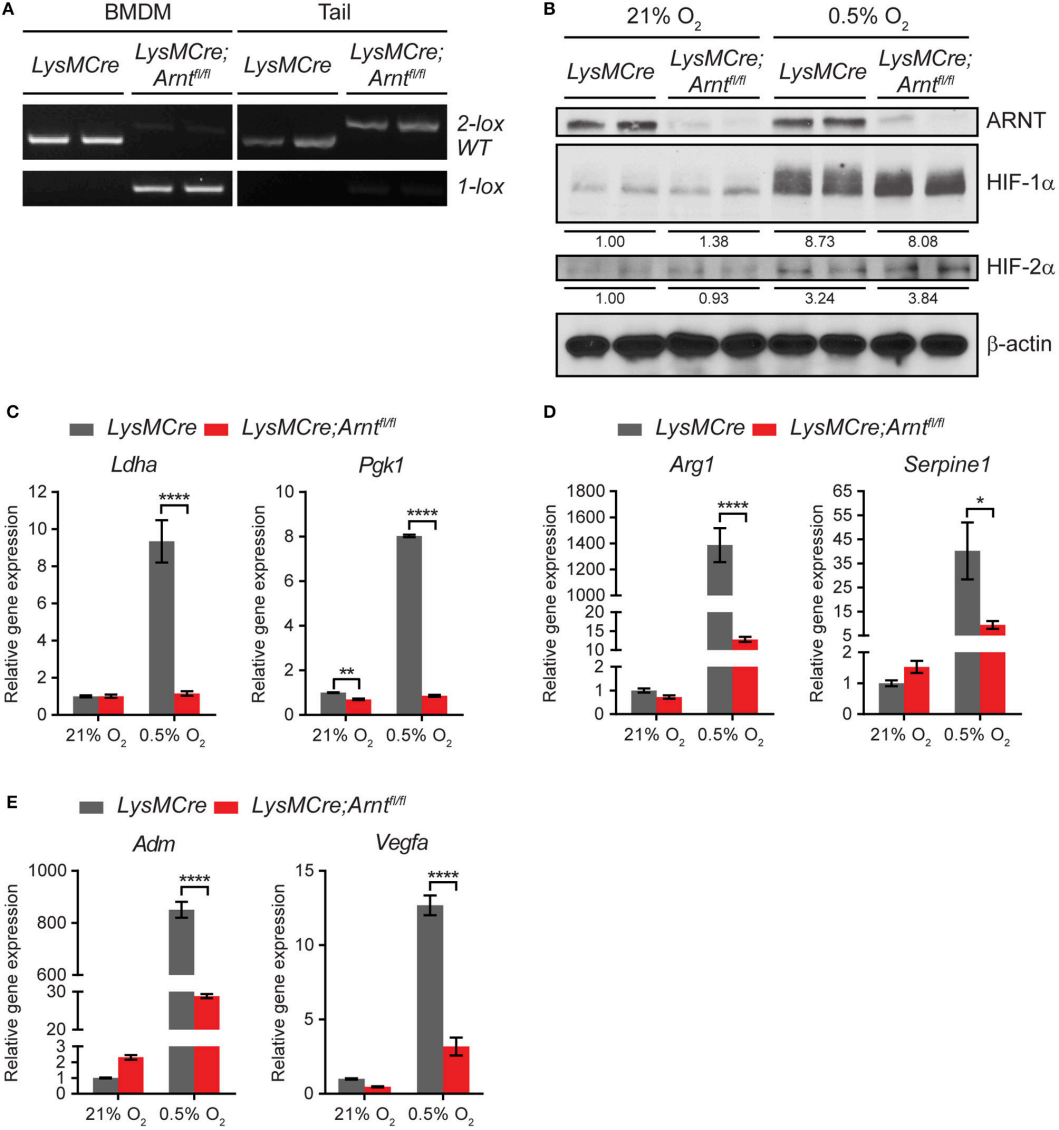
*Arnt* deletion in macrophages disrupts both HIF-1α and HIF -2α transcriptional activity. **(A)** PCR analysis of genomic DNA prepared from bone marrow-derived macrophages (BMDMs) and tail of *LysMCre* and *LysMCre;Arnt*^*fl*/*fl*^ mice. The 2 *lox* band is the floxed allele of *Arnt*, and the 1 *lox* band indicates successful Cre-mediated recombination. **(B)** Western blotting of murine ARNT, HIF-1α, and HIF-2α in protein lysates prepared from *LysMCre* and *LysMCre;Arnt*^*fl*/*fl*^ BMDMs cultured under 21% O_2_ (normoxia) or 0.5% O_2_ (hypoxia) for 24 h. Beta-actin was used as loading control. Densitometry was applied for quantification of HIF-1α and HIF-2α normalized to β-actin. The numbers indicate average values of duplicated samples. **(C–E)** Real-time quantitative PCR (RT-qPCR) analysis of HIF target genes in *LysMCre* (*n* = 3; mean ± s.e.m.) and *LysMCre;Arnt*^*fl*/*fl*^ (*n* = 3; mean ± s.e.m.) BMDMs cultured under 21% O_2_ or 0.5% O_2_ for 24 h. Two-way ANOVA, ^*^p < 0.05, ^**^p < 0.01, and ^****^p < 0.0001.

To study HIF function in myeloid cells, we generated *LysMCre;Arnt*^*fl*/*fl*^ mice to achieve myeloid cell-specific *Arnt* deletion, whereas *LysMCre;Arnt*^+/+^ (henceforth *LysMCre*) mice were used as controls. The *LysMCre* transgene drives efficient deletion of conditional “floxed” alleles in neutrophils and macrophages, which increases as monocytes mature into macrophages (45). Highly specific *Arnt* recombination was confirmed using polymerase chain reaction (PCR) assays to detect the deleted (1 *lox*) *Arnt* allele in DNA isolated from bone marrow-derived macrophages (BMDMs) (lane 3–4), but not tail tissues (lane 7–8), in *LysMCre;Arnt*^*fl*/*fl*^ mice (Figure 1A). ARNT protein abundance was assessed by western blot analysis of lysates from BMDMs cultured under either normoxic (21% O_2_) or hypoxic (0.5% O_2_) conditions for 24 h, at which time ARNT protein was significantly depleted in *LysMCre;Arnt*^*fl*/*fl*^ BMDMs (Figure 1B). Both HIF-1α and HIF-2α were stabilized under hypoxia, irrespective of the presence or absence of ARNT protein (Figure 1B). While not as dramatic as HIF-1α protein stabilization (>8-fold increase), HIF-2α exhibited perceptible accumulation (>3-fold increase) under hypoxic conditions (Figure 1B). The same cells were examined to test whether ARNT depletion was sufficient to abrogate both HIF-1α and HIF-2α transcriptional activity. HIF-1α-specific (*Ldha, Pgk1*), HIF-2α-specific (*Arg1, Serpine1*), and common target genes of both HIF-α subunits (*Adm, Vegfa*) displayed increased transcription in *LysMCre* BMDMs challenged with hypoxia; however, this induction was greatly diminished in *LysMCre;Arnt*^*fl*/*fl*^ BMDMs (Figures 1C–E). Notably, neither phagocytosis nor intracellular ATP levels were significantly affected by ARNT loss in BMDMs (Figures S1A,B), and age-matched *LysMCre* and *LysMCre;Arnt*^*fl*/*fl*^ mice exhibited comparable body weight and fertility (data not shown). Furthermore, no obvious differences were observed in peripheral lymphocyte composition of mouse spleen, including B cells, T cells, macrophages, neutrophils, Ly6C^+^ monocytic cells, and dendritic cells (Figure S1C). Overall, these data indicate that ARNT depletion disrupts both HIF-1α and HIF-2α transcriptional activity in myeloid cells, offering an excellent opportunity to study myeloid pan-HIF inhibition in multiple disease models, including colitis.

### Myeloid deficiency of HIF-α/ARNT heterodimers hinders resolution of DSS-induced acute colitis

**Figure 2:**
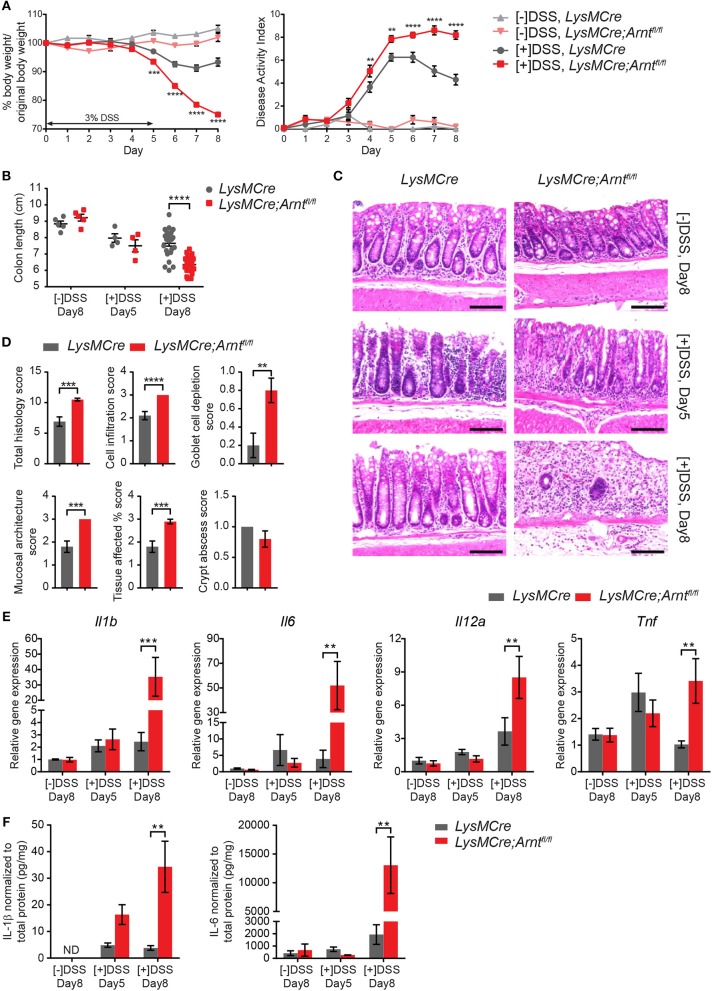
Myeloid HIF-α/ARNT heterodimer deficiency impairs resolution of DSS-induced acute colitis. **(A)** Graphical summary of body weight changes (left panel) and Disease Activity Index (right panel) of *LysMCre* and *LysMCre;Arnt*^*fl*/*fl*^ mice. See Methods for scoring system of Disease Activity Index. Experimental groups (*n* = 24 for each genotype; mean ± s.e.m.) received 3% DSS in drinking water for 5 days, followed by 3 days on regular drinking water. Control groups (*n* = 5 for each genotype; mean ± s.e.m.) received regular drinking water for 8 days. **(B,C)** Colon length **(B)** and H&E images of colon about 1 cm from rectum **(C)** from untreated groups sacrificed on day 8 and DSS-treated groups sacrificed on day 5 and 8. Scale bars, 100 μm. **(D)** Histopathological scoring of colon from mice challenged with DSS for 5 days and sacrificed on day 8. Student's *t*-test. **(E,F)** Following analyzes include untreated group ([–]DSS) sacrificed on day 8 (*n* = 4; mean ± s.e.m.) and DSS-treated groups ([+]DSS) sacrificed on day 5 (*n* = 4; mean ± s.e.m.) and day 8 (*n* = 6; mean ± s.e.m.). **(E)** RT-qPCR analysis of genes encoding pro-inflammatory cytokines from colon tissue. **(F)** ELISA analysis of IL-1β and IL-6 from colonic explant supernatants. Two-way ANOVA was applied for **(A,B,E,F)**, ^**^p < 0.01, ^***^p < 0.001, and ^****^p < 0.0001.

To determine the effects of myeloid ARNT deficiency in a model of acute colitis, *LysMCre* and *LysMCre;Arnt*^*fl*/*fl*^ animals were administered drinking water containing 3% DSS for 5 days, followed by 3 days of regular water. Importantly, mice of both genotypes were housed in the same cages to minimize potential confounding influences from differing microbiomes. Mice were sacrificed on Day 5 ([+]DSS, Day 5) and Day 8 ([+]DSS, Day 8) to compare effects on colitis “induction” and “resolution” phases, which are crucial for pathogen elimination and homeostatic tissue restoration, respectively (15, 53, 54). As expected, *LysMCre* and *LysMCre;Arnt*^*fl*/*fl*^ mice imbibing regular drinking water for 8 days ([–]DSS, Day 8) showed no body weight loss, diarrhea, or fecal blood (collectively represented by “Disease Activity Index” [DAI], see Methods for scoring system) over the course of this experiment (Figure 2A). In contrast, DSS treatment induced colonic inflammation in both *LysMCre* and *LysMCre;Arnt*^*fl*/*fl*^ animals, manifested by decreased body weight, increased DAI, and shortened colon length (Figures 2A,B). Body weight loss and DAI on Day 5 were largely identical in *LysMCre* and *LysMCre;Arnt*^*fl*/*fl*^ mice. Interestingly, as body weight and DAI gradually improved in *LysMCre* mice starting at Day 6, *LysMCre;Arnt*^*fl*/*fl*^ mice continued to exhibit weight loss and an elevated DAI through Day 8 (Figure 2A). Similarly, Day 5 colon lengths were comparable between the two groups; however, *LysMCre;Arnt*^*fl*/*fl*^ mice displayed significantly shorter colons compared to *LysMCre* mice on Day 8 (Figure 2B). Histological evaluation revealed immune cell infiltration and disrupted epithelium on Day 5 in both cohorts (Figure 2C). On Day 8, *LysMCre* mice displayed a colonic histology similar to untreated colons, whereas colons from *LysMCre;Arnt*^*fl*/*fl*^ mice had elevated immune cell filtration and relatively few normal crypt structures (Figure 2C). This difference in histology on Day 8 was confirmed and quantified by histopathological scoring (see Methods; Figure 2D).

RNA expression and secretion of key pro-inflammatory cytokines (IL1β, IL6, IL12α, and TNFα) were also substantially increased in the colon of *LysMCre;Arnt*^*fl*/*fl*^ mice, compared to controls, especially on Day 8 (Figures 2E,F). Beyond IL-6, no dramatic difference in expression of these genes was observed between treated and untreated animals (regardless of genotype) on Day 5, consistent with previous reports (43, 55). Further unbiased cytokine array analysis revealed a more pro-inflammatory microenvironment in *LysMCre;Arnt*^*fl*/*fl*^ colons on Day 8 as well (Figures S2A,B). Enhanced cytokine secretion reflected changes in the expression of corresponding genes (Figure S2C). We also assessed IL-10 and Annexin A1 levels, which are known to exert anti-inflammatory roles during colitis (56, 57, 58), using ELISA and immunohistochemistry, respectively. IL-10 levels were higher in the *LysMCre;Arnt*^*fl*/*fl*^ colons compared to *LysMCre* colons, although the observed changes failed to achieve statistical significance (Figure S2D). Increased IL-10 levels may be a compensatory response of the colon to dampen overly active inflammation, consistent with previous reports demonstrating higher IL-10 levels in more severely damaged colons using the same DSS-induced colitis model (42, 59, 60). Annexin A1, on the other hand, was readily detected in colonic tissue sections, but did not show remarkable differences between control and mutant animals (data not shown). Moreover, eicosanoids and previously described “specialized pro-resolving mediators” (SPMs) (61–65) were analyzed in Day 8 colonic explant supernatants using LC/MS. The levels of SPMs (Resolvin D1, D2, E1, Protectin D1, and Maresin 1) were below the limits of detection. By contrast, leukotriene B4 (LTB_4_), another key mediator of inflammation, was significantly elevated in *LysMCre;Arnt*^*fl*/*fl*^ colons (Figure S2E). Given that LTB_4_ has been shown to exacerbate colitis (66, 67), its increase in *LysMCre;Arnt*^*fl*/*fl*^ colons reinforced the observation that myeloid ARNT deficiency resulted in a more inflamed colonic microenvironment. Collectively, these data suggest that myeloid ARNT is required for proper resolution of acute colitis.

### Loss of either HIF-1α or HIF-2α in myeloid cells phenocopies *LysMCre;Arnt^*fl*/*fl*^* mice in DSS-induced colitis model

It is well-established that ARNT can heterodimerize with the bHLH/PAS transcription factor aryl hydrocarbon receptor (AhR), which also regulates immune responses during intestinal inflammation (68, 69). As expected, increased expression of two AhR target genes was observed in *LysMCre* BMDMs exposed to the AhR agonist (6-formylindolo[3,2-b]carbazole, aka FICZ), but not in *LysMCre;Arnt*^*fl*/*fl*^ BMDMs (Figure S3A). However, ARNT loss had no effect on AhR target gene expression under hypoxic conditions (Figure S3B), suggesting that AhR contributes little to ARNT-dependent responses in hypoxic inflamed colon tissue. Moreover, RNA expression assessment of sorted colonic macrophages failed to reveal changes in AhR target gene expression between the two cohorts (see Figure 6).

**Figure 3:**
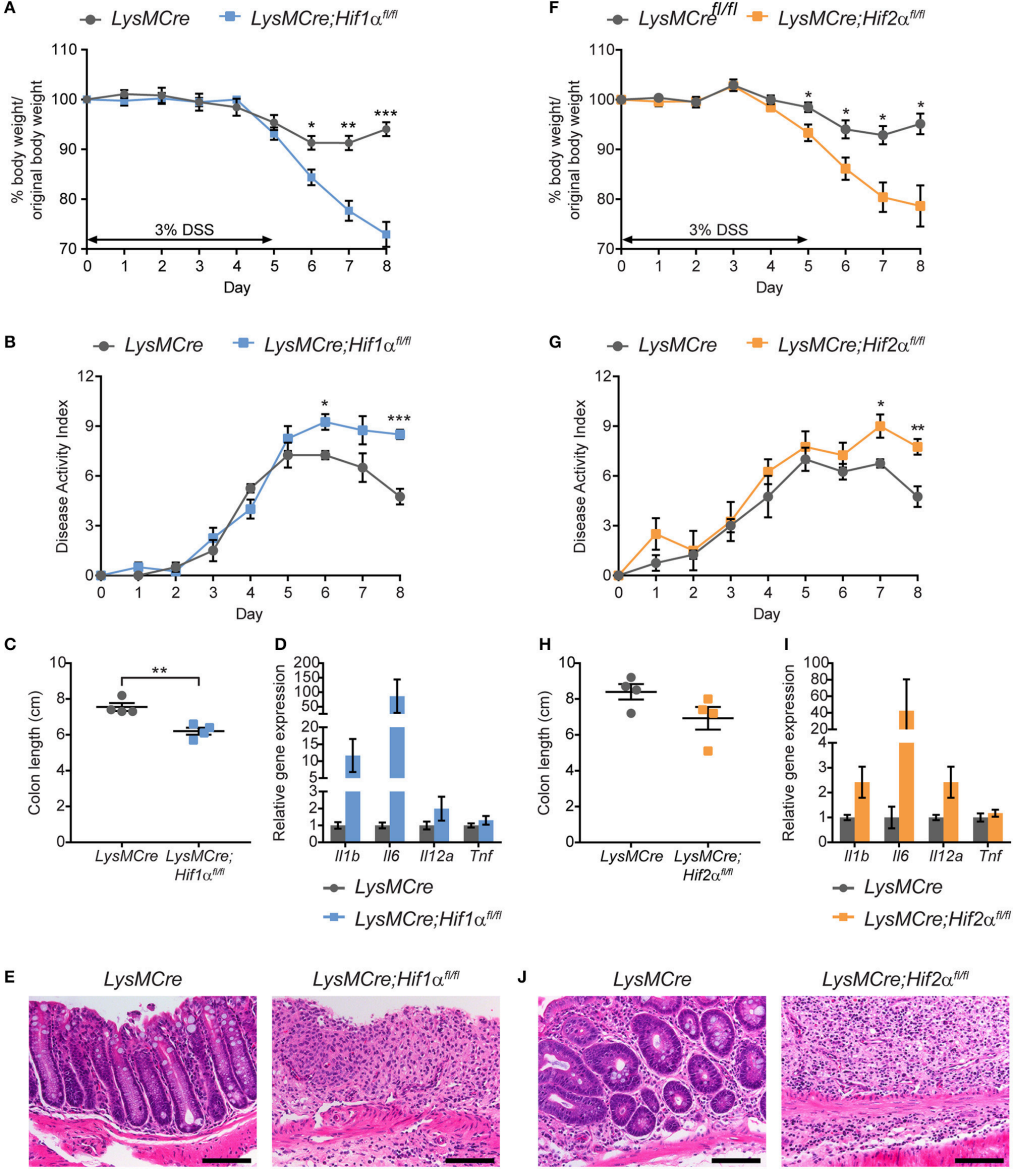
Myeloid HIF-1α and HIF-2α are required for proper resolution of DSS-induced acute colitis. Graphical summary of body weight changes **(A,F)**, Disease Activity Index **(B,G)**, colon lengths **(C,H)**, expression of pro-inflammatory cytokine genes **(D,I)**, and H&E images of colon about 1 cm from rectum **(E,J)** of *LysMCre* (*n* = 4; mean ± s.e.m.) and *LysMCre;Hif1*α^*fl*/*fl*^ mice (*n* = 4; mean ± s.e.m.) or *LysMCre;Hif2*α^*fl*/*fl*^ mice (*n* = 4; mean ± s.e.m.). All mice received 3% DSS in drinking water for 5 days, followed by 3 days on regular drinking water, and sacrificed on day 8. Analyses in **(C–E,H–J)** were performed using colonic tissues from mice sacrificed on Day 8. Student's *t*-test, ^*^p < 0.05, ^**^p < 0.01, and ^***^p < 0.001.

To determine whether myeloid HIF-1α and HIF-2α both contribute to the resolution phase of intestinal inflammation, we induced acute colitis in *LysMCre;Hif1*α^*fl*/*fl*^ and *LysMCre;Hif2*α^*fl*/*fl*^ mice using an identical treatment regimen as that described above (Figure 2). As before, control *LysMCre* mice regained normal body weight after removal of DSS water, whereas *LysMCre;Hif1*α^*fl*/*fl*^ and *LysMCre;Hif2*α^*fl*/*fl*^ animals did not (Figures 3A,F). DAI also remained high following DSS treatment in these mice, compared to *LysMCre* controls (Figures 3B,G). Colon length showed considerable shortening in both HIF-deficient cohorts on Day 8, although the difference between *LysMCre* and *LysMCre;Hif2*α^*fl*/*fl*^ mice failed to reach statistical significance (Figures 3C,H). Elevated gene expression of key pro-inflammatory cytokines (Figures 3D,I) and exacerbated histopathology on Day 8 (Figures 3E,J) in *LysMCre;Hif1*α^*fl*/*fl*^ and *LysMCre;Hif2*α^*fl*/*fl*^ mice further confirmed unresolved inflammation in mice with myeloid HIF-α depletion. We conclude that expression of both HIF-1α and HIF-2α in myeloid cells is critical for resolving acute colonic inflammation. Interestingly, the hypoxic induction of HIF-2α (see Figure 1) and impact of HIF-2α on colitis are less dramatic than HIF-1α (see below for further discussion).

### Myeloid HIF deficiency leads to more neutrophils and Ly6C^+^ monocytic cells in the inflamed colons

**Figure 4:**
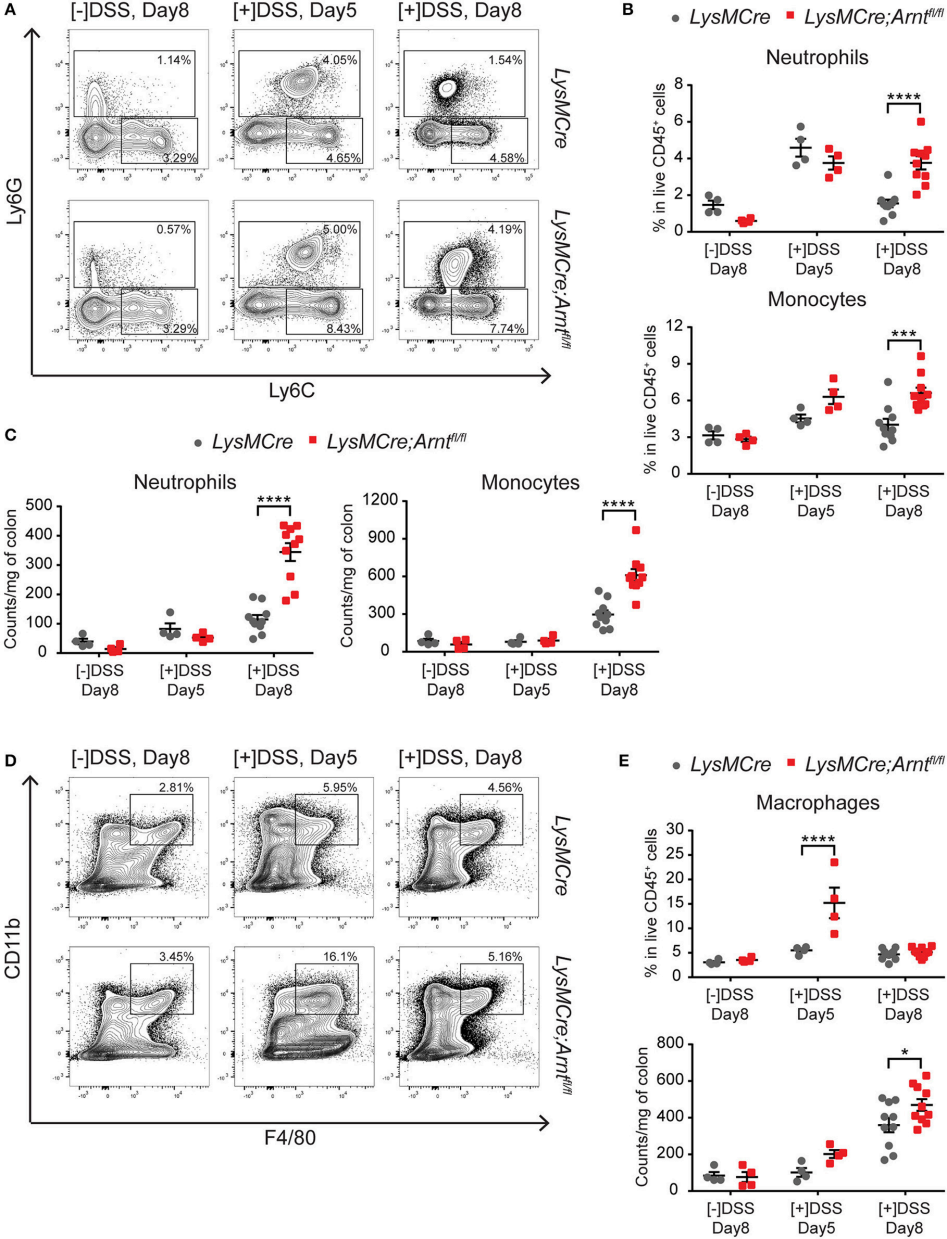
Characterization of myeloid cells in *LysMCre* and *LysMCre;Arnt*^*fl*/*fl*^ lamina propria. Following analyzes include untreated group ([–]DSS) sacrificed on day 8 (*n* = 4) and DSS-treated groups ([+]DSS) sacrificed on day 5 (*n* = 4) and day 8 (*n* = 10). **(A)** Representative gating of neutrophils (Ly6G^+^) and Ly6C^+^ monocytic cells (Ly6G^−^, Ly6C^+^) in live CD45^+^, CD11b^+^, CD11c^−^ cells. **(B)** Percentages of neutrophils (top panel) and Ly6C^+^ monocytic cells (bottom panel) in live CD45^+^ cells. **(C)** Absolute counts normalized to colon weight of neutrophils (left panel) and Ly6C^+^ monocytic cells (right panel). **(D)** Representative gating of macrophages (CD11b^+^, F4/80^+^) in live CD45^+^cells. **(E)** Percentages in live CD45^+^ cells (top panel) and absolute counts normalized to colon weight (bottom panel) of macrophages in lamina propria. Data presented as mean ± s.e.m. Two-way ANOVA, ^*^p < 0.05, ^***^p < 0.001, and ^****^p < 0.0001.

Elevated and persistent immune responses frequently contribute to IBD pathogenesis. To monitor changes in colonic immune cell populations in the context of myeloid HIF disruption, we performed a comprehensive FACS analysis of immune cells recovered from the lamina propria of *LysMCre* and *LysMCre;Arnt*^*fl*/*fl*^ mice treated with DSS. Compared to control *LysMCre* mice, increased numbers of neutrophils (CD45^+^, CD11c^−^, CD11b^+^, Ly6G^+^) and Ly6C^+^ monocytic cells (CD45^+^, CD11c^−^, CD11b^+^, Ly6C^hi^, Ly6G^−^; Figure 4A) were observed in *LysMCre;Arnt*^*fl*/*fl*^ mice on Day 8, expressed either as a percentage of CD45^+^ cells (Figure 4B) or as the number of cells per mg of colon tissue (Figure 4C). DSS treatment elevated neutrophil and monocyte numbers (Figure 4B) in both *LysMCre* and *LysMCre;Arnt*^*fl*/*fl*^ mice on Day 5; however, no significant difference between genotypes was detected. We hypothesized that increased numbers of neutrophils and Ly6C^+^ monocytic cells in colonic tissue of *LysMCre;Arnt*^*fl*/*fl*^ mice could contribute to more severe inflammation, particularly during the resolution phase of acute colitis.

Interestingly, the percentage of macrophages (CD45^+^, CD11b^+^, F4/80^+^), which included resident macrophages and Ly6C^+^MHCII^+^ “maturing macrophages,” (Figure 4D) in CD45^+^ cells was significantly higher in *LysMCre;Arnt*^*fl*/*fl*^ mice than in *LysMCre* mice at Day 5 (Figure 4E). However, by Day 8, the percentage of macrophages between the two groups was comparable, suggesting that HIF deficiency in macrophages could be of particular importance for the shift from the induction phase to the resolution phase of colitis.

As expected, both B and T cells showed no significant differences in the lamina propria of *LysMCre* and *LysMCre;Arnt*^*fl*/*fl*^ mice (Figures S4A,B). Of note, regulatory T cells (Tregs, CD45^+^CD3e^+^CD4^+^CD8a^−^CD25^+^) did not differ between the two cohorts on Day 5 and 8 in the DSS treated animals (Figure S4C). Gene expression of *Il17a* in colonic tissues also suggests that Th17 cell numbers are unlikely to be disrupted by ARNT loss in myeloid cells (Figure S4D). Together, these data indicate that myeloid HIF deficiency doesn't result in a profound change in the lymphoid compartment, consistent with previous data indicating that the DSS-induced colitis model is primarily driven by innate immune cells (70).

### Myeloid HIF deficiency contributes to increased neutrophil numbers primarily through elevated infiltration

Neutrophils often exert pro-inflammatory functions at sites of inflammation (71, 72), and a neutrophil-derived protein, myeloperoxidase, is widely used as a marker of colitis severity in IBD (73–75). Therefore, we investigated the cellular mechanisms contributing to elevated numbers of neutrophils in DSS-treated *LysMCre;Arnt*^*fl*/*fl*^ mice. Given that both neutrophils and macrophages are targeted by *LysMCre* recombination strategies, we first enriched neutrophils from bone marrow (Figures S5A,B) and compared the efficiency of *Arnt* deletion in these two cell types by PCR (Figure S5C). Neutrophils exhibited relatively lower deletion efficiency (≥60%) compared to macrophages (≥80%; Figure S5D), which correlated to decreases in *Arnt* mRNA levels (Figure S5E). Nevertheless, *Arnt* deletion in neutrophils was sufficient to disable hypoxic induction of *Vegfa* gene expression in *LysMCre;Arnt*^*fl*/*fl*^ BMDNs (Figure S5F). We next determined if neutrophils were affected by cell-intrinsic factors, cell-extrinsic factors, or a combination of the two.

**Figure 5:**
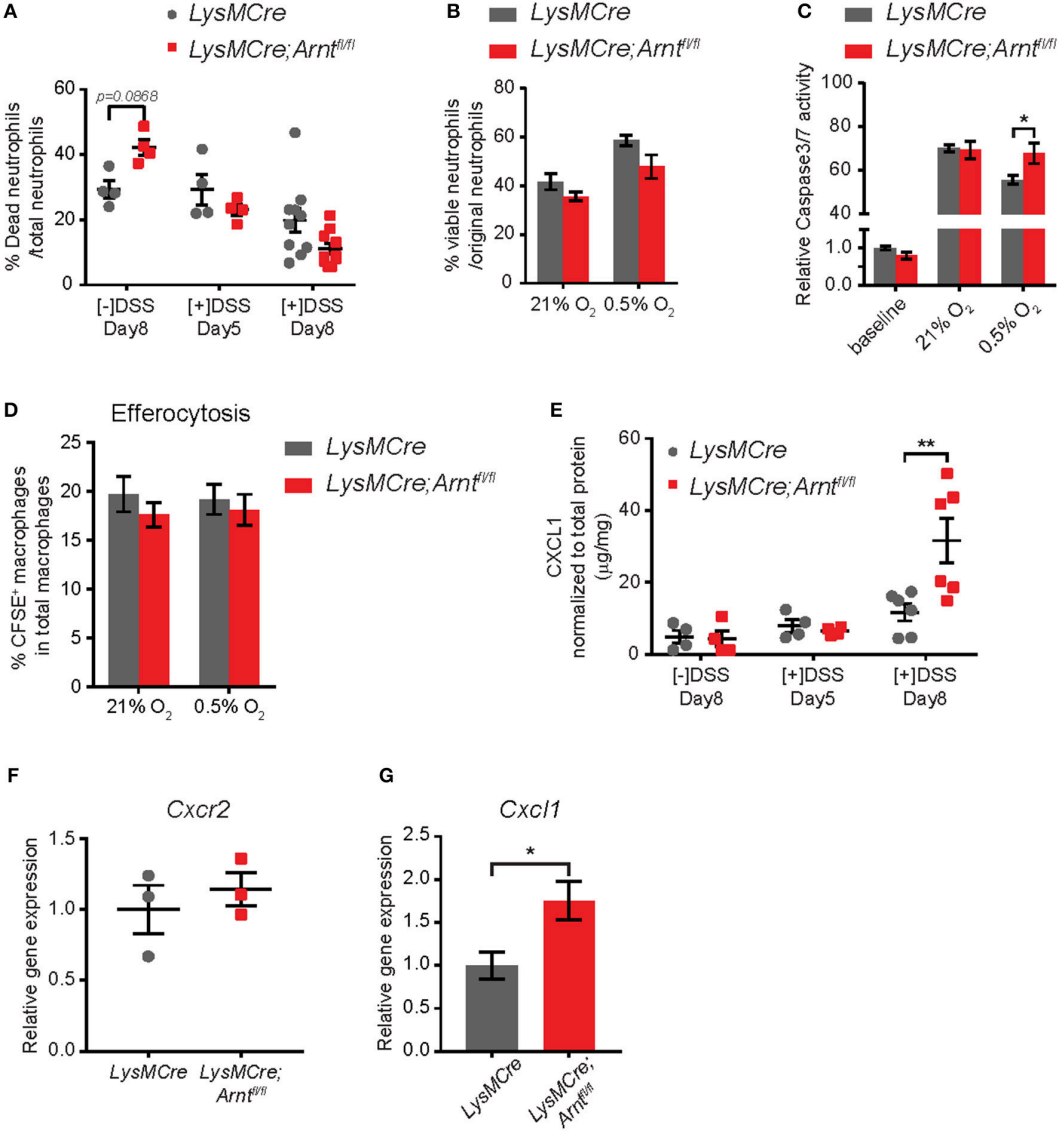
Increased neutrophil numbers in lamina propria of *LysMCre;Arnt*^*fl*/*fl*^ mice is due to elevated CXCL1, not enhanced survival. **(A)** Percentage of dead neutrophils normalized to total neutrophil numbers in lamina propria. Data represent untreated mice ([–]DSS) sacrificed on day 8 (*n* = 4) and DSS-treated mice ([+]DSS) sacrificed on day 5 (*n* = 4) and day 8 (*n* = 10). **(B)** Viability of bone marrow-derived neutrophils (BMDNs) from *LysMCre* (*n* = 3) and *LysMCre;Arnt*^*fl*/*fl*^ (*n* = 3) mice cultured under normoxia or hypoxia for 24 h. Viability was determined by Trypan Blue exclusion. **(C)** Caspase3/7 activity assay performed with BMDNs from *LysMCre* (*n* = 3) and *LysMCre;Arnt*^*fl*/*fl*^ (*n* = 3) mice. Baseline measurement was conducted using BMDNs immediately after isolation from bone marrow. **(D)** Efferocytosis of apoptotic neutrophils by *LysMCre* (*n* = 3) and *LysMCre;Arnt*^*fl*/*fl*^ (*n* = 3) BMDMs under normoxia and hypoxia overnight (14 h). CFSE^+^ macrophages represent macrophages that engulfed CFSE-labeled apoptotic neutrophils. **(E)** ELISA analysis of CXCL1 in colonic explant supernatants. **(F)** RT-qPCR analysis of *Cxcr2* expression in *LysMCre* and *LysMCre;Arnt*^*fl*/*fl*^ BMDNs. **(G)** RT-qPCR analysis of *Cxcl1* expression in sorted lamina propria macrophages from *LysMCre* and *LysMCre;Arnt*^*fl*/*fl*^ mice. Data presented as mean ± s.e.m. Two-way ANOVA for **(A–E)** and Student's *t*-test for **(F,G)**; ^*^p < 0.05 and ^**^p < 0.01.

Timely neutrophil apoptosis is a key event that initiates the resolution of acute inflammation (15, 76–78). It was therefore plausible that the increase in neutrophil numbers during the resolution phase was due to delayed apoptosis; however, given previously described pro-survival functions of both HIF-1α and HIF-2α in neutrophils (30, 31), this seemed unlikely. Consistent with these observations, untreated *LysMCre;Arnt*^*fl*/*fl*^ mice exhibited decreased neutrophil viability (Figure 5A). Furthermore, the percentage of dead neutrophils in colon tissue from *LysMCre* and *LysMCre;Arnt*^*fl*/*fl*^ mice was indistinguishable at Day 5 and 8 (Figure 5A). *In vitro*, the percentage of viable BMDNs after 24-hour culture under normoxia or hypoxia were also comparable between *LysMCre* and *LysMCre;Arnt*^*fl*/*fl*^ cohorts (Figure 5B). Caspase 3/7 activity assessment further supported the notion that ARNT deficiency can promote, as opposed to delay, neutrophil apoptosis under hypoxia (Figure 5C), consistent with previous findings (30, 31). Apoptotic neutrophil removal by macrophages is necessary for an efficient resolution program (15, 76). We therefore examined macrophage ability to efferocytose apoptotic neutrophils. As shown in Figure 5D, efferocytosis by BMDMs under normoxia and hypoxia was not disrupted by myeloid HIF deficiency, suggesting other factors contribute to higher neutrophil numbers in *LysMCre;Arnt*^*fl*/*fl*^ colons.

Another critical step in resolving inflammation is the prevention of further neutrophil recruitment (15, 54, 76). We therefore tested whether myeloid HIF deficiency enhanced neutrophil infiltration by altering either the microenvironment or neutrophil chemotaxis. CXCL1 has long been recognized as a major neutrophil chemoattractant, and CXCL1 secretion in the supernatant of colonic explants was increased by almost 3-fold in *LysMCre;Arnt*^*fl*/*fl*^ mice on Day 8, compared to *LysMCre* mice (Figure 5E). In contrast, the levels of CXCR2, the CXCL1 receptor expressed by neutrophils (71), were not affected by ARNT status (Figure 5F), suggesting that the increased number of neutrophils may reflect the elevated secretion of chemoattractant in the gut, rather than enhanced neutrophil migratory ability. Given that CXCL1 production by macrophages has been shown to promote neutrophil infiltration in a peritonitis model (79), we next asked if HIF deficiency promoted CXCL1 production by macrophages. Expression of *Cxcl1* was indeed higher in macrophages sorted from the lamina propria of *LysMCre;Arnt*^*fl*/*fl*^ mice, suggesting that macrophages could be a major source of CXCL1 in the intestine (Figure 5G).

### HIF-deficient colonic macrophages have a diminished pro-resolving profile

**Figure 6:**
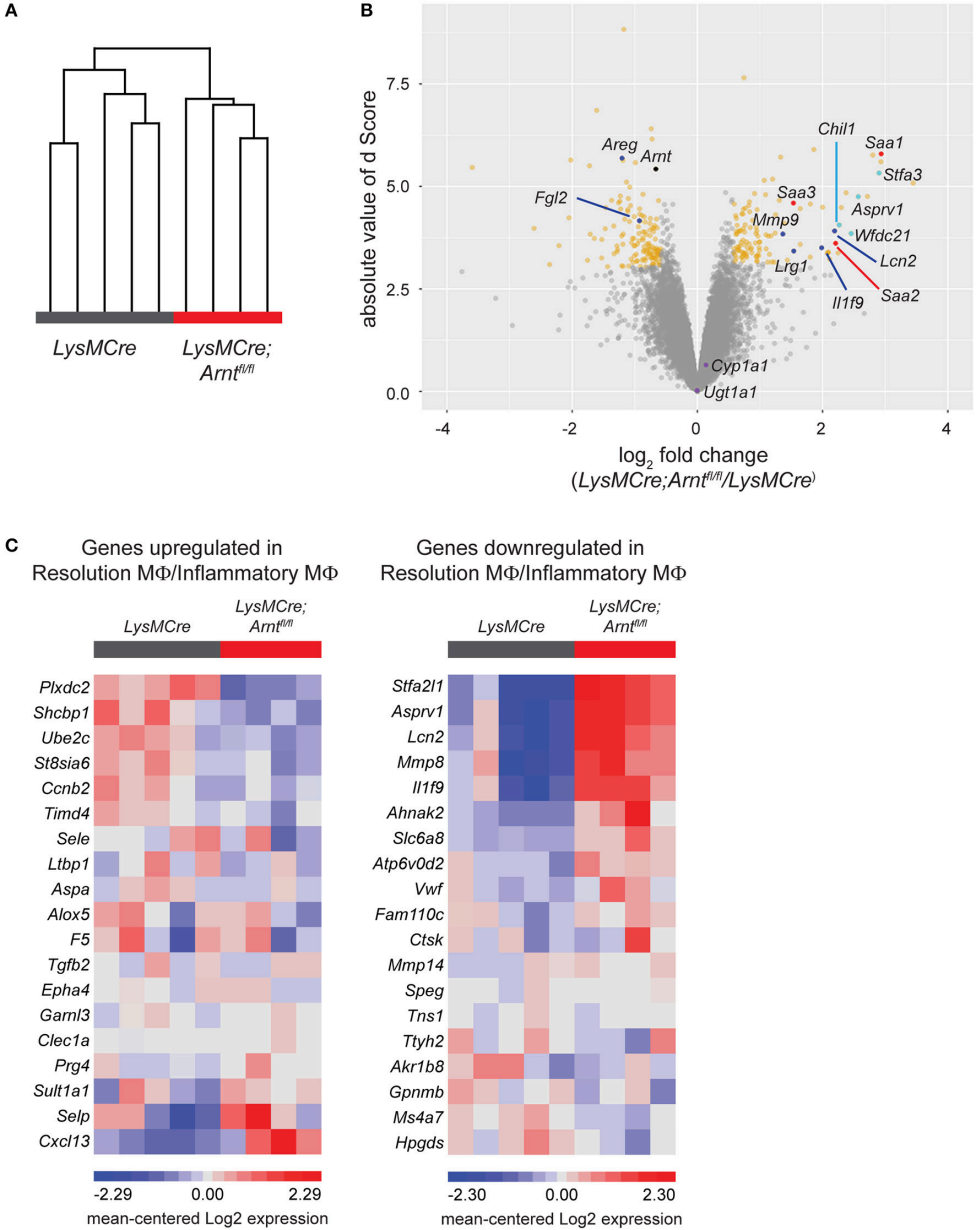
HIF deficiency renders macrophages less pro-resolving. **(A)** Unsupervised hierarchical clustering of microarray samples collected as sorted lamina propria macrophages from *LysMCre* and *LysMCre;Arnt*^*fl*/*fl*^ mice. Each sample was from a single mouse. Clustering was performed on log 2 normalized gene intensities (from robust multi-array average). Average linkage was used with Pearson dissimilarity as the distance measure. **(B)** Volcano plot of the statistical significance (d score, the T-statistic value used in Significance Analysis of Microarrays) against the log 2 ratio of gene expression between lamina propria macrophages from *LysMCre* and *LysMCre;Arnt*^*fl*/*fl*^ mice, based on the microarray analysis. The magnitude of d score scales with statistical significance. Genes with fold change below or above 1.5 and a false discovery rate (*q*-value) smaller than 5% are in yellow. **(C)** Heat maps of gene expression using the top 20 upregulated (left) and downregulated (right) genes in resolution phase macrophages (Gene Express, E-MEXP-3189). Gene expression data are from our microarray analysis of *LysMCre;Arnt*^*fl*/*fl*^ vs. *LysMCre* lamina propria macrophages. Each panel in **(C)** displays 19/20 genes differentially expressed (80), as one gene from each list was not detected in our microarray analysis.

**Figure 7:**
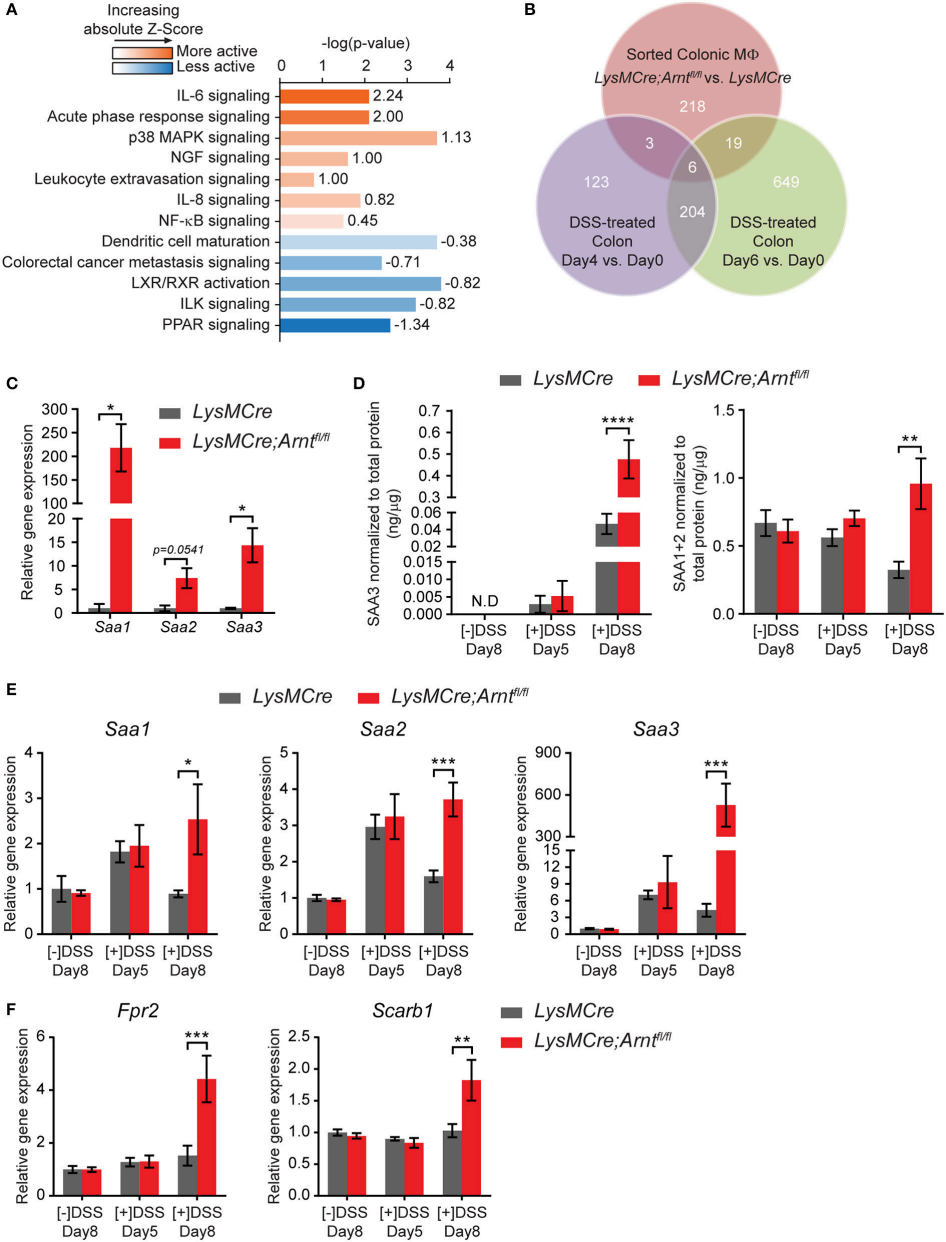
Myeloid HIF deficiency results in increased serum amyloid As production in the colon. **(A)** Ingenuity Pathway Analysis (IPA) of canonical pathways differentially expressed between *LysMCre* (*n* = 5) and *LysMCre;Arnt*^*fl*/*fl*^ (*n* = 4) lamina propria macrophages based on microarray. Numbers on the right end of each bar are activation Z-Score of that pathway. A positive Z-Score (colored orange) indicates likely activation of that pathway in *LysMCre;Arnt*^*fl*/*fl*^ compared to *LysMCre* lamina propria macrophages, and a negative Z-Score (colored blue) indicates likely inactivation. Z-Score > 2 or < −2 is considered significant. **(B)** Venn diagram showing overlapping between differentially expressed genes in (red circle) *LysMCre* vs. *LysMCre;Arnt*^*fl*/*fl*^ lamina propria macrophages (absolute fold change >1.5 and *q*-value < 5%), (purple circle) Day 4 vs. 0 and (green circle), and Day 6 vs. 0 DSS-treated colon (absolute fold change > 2 and *q*-value < 5%) based on GSE22307. **(C)** RT-qPCR analysis of *Saa1, Saa2*, and *Saa3* in sorted lamina propria macrophages from mice challenged with DSS for 5 days and sacrificed on Day 8. Data presented as mean ± s.e.m. Student's *t*-test, ^*^p < 0.05. **(D–F)** Data represent untreated mice ([–]DSS) sacrificed on day 8 (*n* = 4) and DSS-treated mice ([+]DSS) sacrificed on day 5 (*n* = 4) and day 8 (*n* = 6). **(D)** ELISA analyzes of SAA3 (left panel) and SAA1/2 (right panel) in colonic explant supernatants. **(E,F)** RT-qPCR analysis of **(E)** *Saa1, Saa2*, and *Saa3* and **(F)** *Fpr2, Scarb1* in colon tissues. Data presented as mean ± s.e.m. Two-way ANOVA for **(D–F)**; ^*^p < 0.05, ^**^p < 0.01, ^***^p < 0.001, and ^****^p < 0.0001.

To overcome inflammation, macrophages must activate “pro-resolving” functions to ensure reconstitution of tissue homeostasis (15, 76). To elucidate the exact contribution of HIF-deficient macrophages to unresolved colitis, we sorted these cells in colitic tissues from *LysMCre* (*n* = 5) and *LysMCre;Arnt*^*fl*/*fl*^ (*n* = 4) mice on Day 8, and conducted RNA microarray analysis. Unsupervised clustering clearly distinguished the two cohorts, indicating similar gene expression patterns among mice with the same genotype (Figure 6A). Together, 115 upregulated and 138 downregulated genes were identified in *LysMCre;Arnt*^*fl*/*fl*^ compared to *LysMCre* macrophages, based on a minimum 1.5X absolute change cutoff and a false discovery rate (FDR) adjusted *p*-value (stated as *q*-value here) of 0.05 (Figure 6B, yellow dots). Decreased *Arnt* gene expression was also verified (Figure 6B, black dot). Expression of multiple genes previously associated with adverse effects in IBD, including *Lcn2, Il1f9, Lrg1*, and *Mmp9* (Figure 6B, blue dots), was higher in *LysMCre;Arnt*^*fl*/*fl*^ macrophages. Simultaneously, several genes whose products act to limit or resolve colitis [e.g., *Areg and Fgl2* (Figure 6B, blue dots)] showed lower expression levels in HIF-deficient macrophages. We compared our microarray results with a previous genome profiling study performed over the time course of DSS-induced colitis (55). Twenty-eight genes exhibited overlap between both datasets (see Figure 7B), which included *Lcn2 and Lrg1*, pro-inflammatory genes previously implicated in colitis. Of note, expression of two key target genes of AhR signaling (*Cyp1a1 and Ugt1a1*) was not significantly altered in ARNT-deficient intestinal macrophages (Figure 6B, purple dots), further supporting the notion that AhR signaling is not a significant contributing factor to the phenotypes we observed.

Given the defective resolution of colitis observed in *LysMCre;Arnt*^*fl*/*fl*^ mice, we next asked whether conversion to a pro-resolving phenotype was impaired in HIF-deficient macrophages. Transcriptomic profiles of inflammatory macrophages and resolution phase macrophages have been described in a previous study (80). By comparing differentially expressed genes in *LysMCre;Arnt*^*fl*/*fl*^ and *LysMCre* macrophages to the published differentially expressed genes in inflammatory- and resolution-phase macrophages, we observed that ~70% of the genes in our list were consistent with a reduction in a pro-resolving phenotype of macrophages in *LysMCre;Arnt*^*fl*/*fl*^ mice. For example, of the 20 most highly upregulated genes in resolution macrophages, 13 (65%) were instead *downregulated* in sorted *LysMCre;Arnt*^*fl*/*fl*^ macrophages (Figure 6C, left panel). Similarly, 13 (65%) of the 20 most downregulated genes in resolution macrophages were instead *upregulated* in sorted *LysMCre;Arnt*^*fl*/*fl*^ macrophages (Figure 6C, right panel). Expression of *Alox15*, whose upregulation is a hallmark of pro-resolving macrophages (80, 81), showed a −1.38-fold change (*q*-value = 62.6) in *LysMCre;Arnt*^*fl*/*fl*^ lamina propria macrophages compared to *LysMCre* macrophages, confirmed with RT-qPCR (data not shown). Collectively, these data suggest a critical role of HIF signaling for the functional conversion of macrophages, which might underlie the successful shift from induction phase to resolution phase in acute colitis.

Multiple studies suggest that macrophage “polarization” into M1 or M2 fates can regulate a variety of inflammatory conditions (82–88). To test if macrophage polarization was affected by *Arnt* deletion, we cultured *LysMCre* and *LysMCre;Arnt*^*fl*/*fl*^ BMDMs under either normoxia or hypoxia, with or without stimuli that induce an M1 (5 ng/mL LPS+1 ng/mL IFNγ) or M2 (5 ng/mL IL-4+5 ng/mL IL13) phenotype. Based on qPCR analysis of several canonical M1- and M2-associated markers, we found that M1 polarization was enhanced (Figure S6A), whereas M2 polarization was suppressed (Figure S6B). In contrast, when microarray data of sorted macrophages recovered from colon tissue were subject to a comprehensive analysis of M1 and M2 markers, we observed a mixed profile of polarization, marked by repression of both M1 and M2 gene signatures (Figures S6C,D). Consequently, it remains unclear from these data whether myeloid HIF deficiency favors one polarization state over the other *in vivo*.

### Myeloid HIF deficiency increases serum amyloid a levels in the colon

Among the differentially regulated genes displayed in Figure 6B, those encoding three members of serum amyloid A (SAA) family were identified among the most upregulated genes in *LysMCre;Arnt*^*fl*/*fl*^ macrophages (red dots). Ingenuity Pathway Analysis (IPA) also identified acute phase response signaling as one of the most activated signaling pathways in *LysMCre;Arnt*^*fl*/*fl*^ macrophages, which includes *Saa1* and *Saa3* (Figure 7A). Comparison of our microarray results with the temporal genome profiling study of colitis (55) revealed *Saa3* as one of the 6 common genes differentially expressed throughout the time course of disease (Figure 7B), implicating a potential role for *Saa3* in this disease model. SAA is an acute phase response protein whose elevated production is often observed in Crohn's disease and other inflammatory conditions (89–92). Moreover, SAA has been implicated in mediating defective resolution by competing with lipoxin A_4_, a key pro-resolving molecule, for binding to their common receptor, formyl peptide receptor 2 (FPR2) (93, 94). Upregulation of these genes were first confirmed using qRT-PCR (Figure 7C). Moreover, supernatants from colonic explants from *LysMCre;Arnt*^*fl*/*fl*^ mice, contained higher levels of SAA1/2 and SAA3 proteins, particularly on Day 8, compared to controls (Figure 7D). Expression of two key SAA receptors (*Fpr2 and Scarb1*) was also elevated in the colon tissue of *LysMCre;Arnt*^*fl*/*fl*^ mice (Figure 7F), reinforcing a potential role for SAA in colitis. We also assessed *Saa* gene expression in colon tissue and found that the trend of *Saa* expression correlated with disease progression (Figure 7E). After 5-days DSS treatment, both *LysMCre* and *LysMCre;Arnt*^*fl*/*fl*^ mice exhibited increased *Saa* expression compared to untreated mice. On Day 8, *Saa* gene expression in *LysMCre* mice declined to a level similar to untreated mice; however, *LysMCre;Arnt*^*fl*/*fl*^ mice maintained a high level of *Saa* gene expression, and an elevated level of *Saa3*. These results again implicate SAA in the regulation of colitis resolution.

KEGG pathway analysis of significantly upregulated genes in *LysMCre;Arnt*^*fl*/*fl*^ lamina propria macrophages suggested changes in arachidonic acid metabolism (Figure S7A), especially increased *Ptges1, Cyp2e1* and *Ggt1* expression (Figure S7B). *Ptges1* encodes prostaglandin E synthase 1 (PTGES1), which catalyzes the production of prostaglandin E2 (PGE_2_) (95). Since PGE_2_ elicits diverse functions during inflammation, we primarily assessed PGE_2_ production and key enzymes responsible for it. *Ptges1* and *Ptges2* upregulation were confirmed using RT-qPCR (Figure S7C). Interestingly, *LysMCre;Arnt*^*fl*/*fl*^ mice exhibited lower levels of PGE_2_ as compared with *LysMCre* mice in the gut (Figure S7D). Further measurement of PGE_2_ in BMDM culture supernatants suggested that decreased PGE_2_ production by HIF-deficient macrophages may partially contribute to these observations in the colon (Figure S7E, left). Of note, PGE_2_ generation in BMDNs was independent of HIF-α/ARNT heterodimers (Figure S7E, right). Decreased *Ptgs2* expression (Figure S7C), encoding cyclooxygenase 2 (COX2), in *LysMCre;Arnt*^*fl*/*fl*^ lamina propria macrophages may be responsible for lower PGE_2_ levels, despite *Ptges1* and *Ptges2* upregulation which are downstream of COX2 in PGE_2_ production. Given PGE_2_ has been shown to facilitate resolution of colonic inflammation (96–98), lower levels of PGE_2_ may partially contribute to defective resolution in *LysMCre;Arnt*^*fl*/*fl*^ mice. However, we note differences of colonic PGE_2_ levels between *LysMCre* and *LysMCre;Arnt*^*fl*/*fl*^ mice are modest, suggesting other factors are important as well.

## Discussion

**Figure 8:**
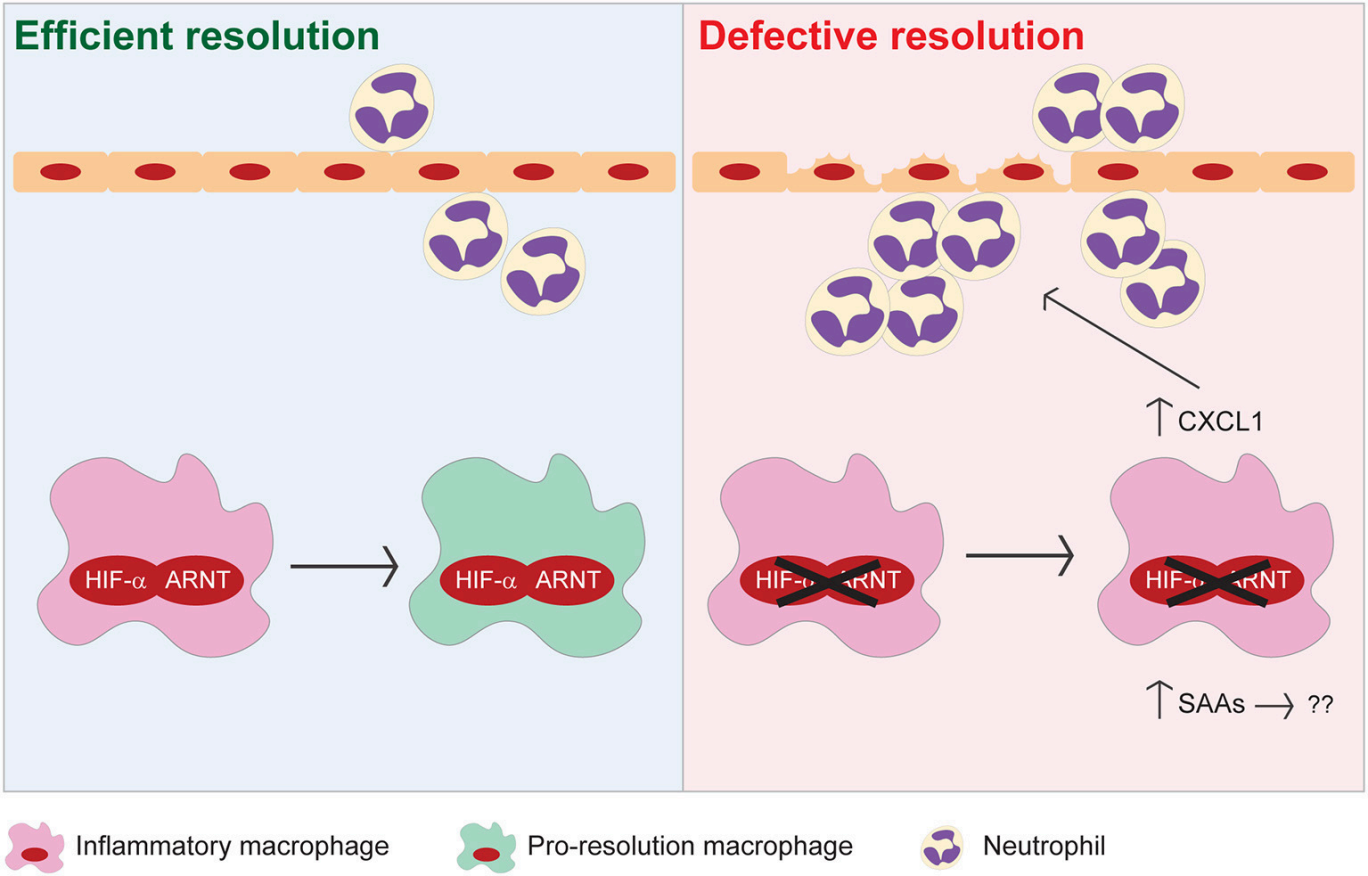
Proposed model illustrating the pro-resolving functions of myeloid HIF-α/ARNT heterodimer during resolution of intestinal inflammation.

In the present study, we demonstrate that HIF signaling in myeloid cells is essential for resolution of acute colitis (Figure 8). Myeloid HIF deficiency increases the infiltration of pro-inflammatory neutrophils and monocytic cells, impedes the functional conversion of macrophages to a pro-resolving phenotype, and promotes SAA production in the colon during the resolution phase. Collectively, these disruptions contribute to defective resolution of colitis in mice with myeloid HIF deficiency.

Previously, pharmacological approaches to stabilize HIFα proteins using PHD inhibitors were shown to suppress intestinal inflammation (37, 38). In line with these findings, our study demonstrates that myeloid HIF function is necessary for proper and timely resolution of acute colitis, highlighting myeloid cells as active contributors to the anti-inflammatory effects of PHD inhibitors. In contrast, a previous study shows that depletion of myeloid ARNT dampens cutaneous inflammation (99). The apparent discrepancy between that study and ours is likely explained by the differences in disease models. Given the plasticity of macrophages, it is not surprising for myeloid HIF to elicit different functions in response to distinct microenvironments in various types of inflammation. For example, we found that myeloid ARNT is dispensable for animal survival in an LPS-induced endotoxemia model and macrophage recruitment in thioglycollate-induced peritoneal inflammation (data not shown). These results strongly suggest that potential pharmacological approaches to stabilize HIF should be selected carefully to match the cellular hypoxic responses in specific diseases.

The importance of HIF signaling in distinct cell types, including epithelial cells (4, 12, 40, 41), dendritic cells (42) and T cells (59), has been studied in the context of intestinal inflammation. We add to these findings by demonstrating that myeloid HIF-1α and HIF-2α are crucial to resolve acute colitis. Although myeloid HIF-1α has been recently implicated in promoting DSS-induced colitis (43, 44), we hypothesize that these disparate results are likely due to different phases of inflammation under examination, and/or different effects of the gene promoters driving Cre-mediated recombination. Specifically, Bäcker and colleagues (43) examined mice up to Day 6 of DSS treatment without time for recovery, as opposed to our study where the principal phenotype was captured after removal of DSS H_2_O. Moreover, we utilized *LysMCre* which targets mature macrophages and Gr-1^+^ myeloid cells, while Kim and colleagues (44) chose *hMRP8Cre* for recombination primarily in Gr-1^+^ granulocytes. These reports underscore the complexity of colitis, and roles of different myeloid populations. Moreover, we note the less impressive contribution of HIF-2α, compared with HIF-1α, to colitis resolution (Figure 3), which may be due to its less dramatic induction under hypoxia in macrophages (Figure 1). We conclude that HIF-1α is the major isoform driving proper resolution of intestinal inflammation, with HIF-2α contributing to a lesser extent.

Previous studies have described a protective role for AhR, another established ARNT binding partner, in colitis (100–102). However, AhR was globally deleted in these mice. Our findings suggest that myeloid AhR is not likely a major contributor to colitis resolution, as disruption of HIF-1α and HIF-2α signaling accounts for the majority of ARNT-dependent effects in our model. Microarray analysis of sorted lamina propria macrophages supports our contention that myeloid AhR signaling is not a significant factor in this intestinal inflammation model. Furthermore, Chinen and colleagues (103) reported that myeloid AhR is dispensable for inflammatory response in DSS-induced colitis by utilizing the same *LysMCre* strategy.

Temporally ordered apoptosis and clearance of neutrophils signals the initiation of inflammation resolution (15, 76), and we observed that unresolved colitis in *LysMCre;Arnt*^*fl*/*fl*^ mice was associated with elevated numbers of neutrophils in the lamina propria (Figure 4), correlating with enhanced expression of chemotactic signals, as opposed to increased neutrophil lifespan (Figure 5). Based on previous understanding that either HIF-1α or HIF-2α prevents neutrophil apoptosis under hypoxia (30, 31), we anticipated that neutrophil viability would be impaired upon *Arnt* deletion; however, this was not observed. We speculate that increased SAA and LTB_4_ in the colonic microenvironment counterbalance the effects of ARNT loss on neutrophil viability, given that SAA and LTB_4_ have been shown to inhibit neutrophil apoptosis (104–107).

The precise roles of SPMs, particularly endogenous SPMs, during inflammation have been debated. Administration of exogenous SPMs clearly prevents intestinal inflammation in multiple murine colitis models (61, 62, 64, 65). On the other hand, other reports found little evidence for endogenous SPM production *in vivo* (108, 109). In the present study, we were unable to detect appreciable levels of SPMs in colonic explant supernatants, regardless of genotype. We conclude that defective resolution of colitis in mice deficient of myeloid HIF signaling is more likely due to factors (e.g., SAAs, CXCL1, and LTB_4_) other than SPMs. Nevertheless, the therapeutic potential of exogenous SPMs in IBD treatment remains an interesting and important field of investigation.

A “partnership” between neutrophils and macrophages is common during inflammation (53), particularly in its resolution phase (15). Here, we suggest that HIF-deficient macrophages promote neutrophil chemotaxis via elevated CXCL1 secretion. As such, we speculate that HIF signaling plays a more dominant role in macrophage functions compared to neutrophils in this setting. In line with this, production of PGE_2_ (Figure S7E), together with other eicosanoid metabolites (e.g., prostaglandin F2α and thromboxane B2; data now shown), is dependent on HIF signaling in macrophages, but not in neutrophils. However, we cannot exclude the possibility that other neutrophil-intrinsic properties are altered upon the ARNT loss. This will be investigated in future studies.

Macrophages adopt a pro-resolving phenotype to facilitate recovery from inflammation (15, 76). Detailed molecular mechanisms underlying this functional macrophage conversion have yet to be elucidated. Here, we uncover a requirement for intact HIF signaling for macrophages to adopt a pro-resolving phenotype in inflamed colons. Similar to a previous report that resolution phase macrophages display neither canonical M1 nor M2 markers (80), our study reveals that disruption of macrophage HIF signaling impedes conversion to a pro-resolving phenotype, without promoting either M1 or M2 identities *in vivo*. This could be due to the highly complex and dynamic microenvironment of colitis. Our findings are consistent with previous studies demonstrating that M1 and M2 polarization is more readily conferred in cell culture settings, which do not necessarily reflect *in vivo* conditions (110).

Microarray analysis of colonic macrophages revealed a link between HIF signaling and *Saa* expression in macrophages. An initial attempt to rescue abnormal resolution in *LysMCre;Arnt*^*fl*/*fl*^ mice by injecting anti-SAA neutralizing antibodies (111) was unsuccessful due to technical complications (data not shown). Given the dearth of validated, commercially available neutralizing antibodies for murine SAA, subsequent work will focus on generating a genetic deletion of *Saa1/2/3* in myeloid cells; however this is beyond the scope of the current study. Furthermore, although SAA function has been implicated previously in regulating inflammation, other genes upregulated in both colitic tissue and *LysMCre;Arnt*^*fl*/*fl*^ macrophages, including *Stfa3, Asprv1, Wfdc21*, and *Chil1* (Figure 6B), may also contribute important functions.

In conclusion, we identify myeloid cells as essential contributors to the protective effects of HIF activation during colitis, and describe an important role for myeloid HIF signaling in the efficient resolution of intestinal inflammation. Myeloid cells present as an attractive target cell population for approaches to engage HIF signaling, such as using PHD inhibitors, as a way to more effectively bring IBD under control.

## Author contributions

NL and MS designed the study. NL, HX, DL, NS, QT, AA, and HM performed the experiments. NL and JS generated the mouse colonies. DL and AA maintained the mouse colonies. NL, HX, and ZZ analyzed the data. NL and MS wrote the manuscript. HM and GF performed the eicosanoid analysis by LC/MS and provided critical technical and scientific guidance and discussion. HW provided anti-SAA neutralizing antibodies.

### Conflict of interest statement

The authors declare that the research was conducted in the absence of any commercial or financial relationships that could be construed as a potential conflict of interest.
