# Supplementary material for: Myeloid Cell Hypoxia-Inducible Factors Promote Resolution of Inflammation in Experimental Colitis
Source: Front Immunol. 2018 Nov 5;9:2565. doi: 10.3389/fimmu.2018.02565 (PMC6230677; doi:10.3389/fimmu.2018.02565)
Supplement: Supplementary file 1 [file Image_1.pdf]

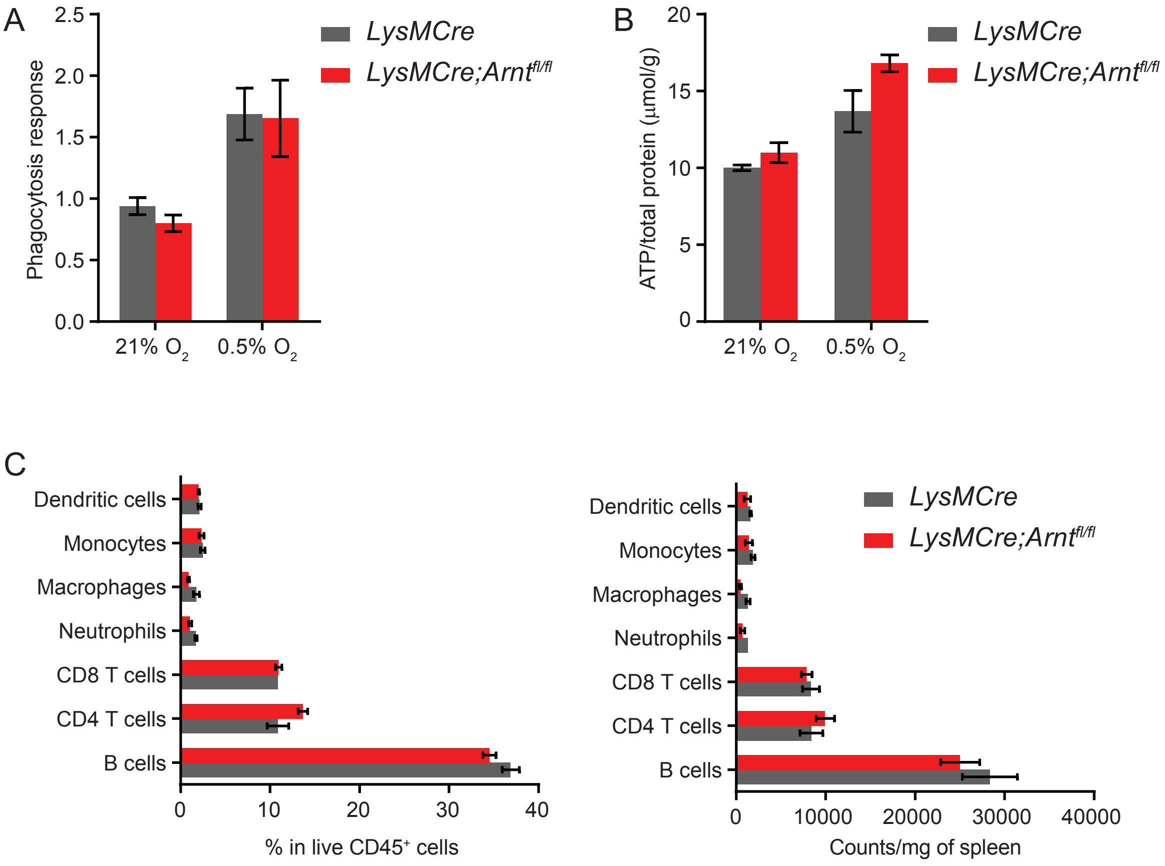

Supplementary Figure S1. Myeloid ARNT depletion does not affect phagocytosis, ATP generation in BMDMs, and peripheral lymphocytes composition. **(A-B)** Phagocytosis response **(A)** and ATP generation normalized to total protein **(B)** of BMDMs cultured under normoxia or hypoxia for 24 hours (n=3; mean ± s.e.m.). Phagocytosis response was measured using Vybrant Phagocytosis Assay Kit (Thermo Fisher Scientific). ATP levels were determined using ATPlite Luminescence ATP Detection Assay System (PerkinElmer). **(C)** Percentage in $CD45^+$ cells (left panel) and absolute cell counts of major immune cell populations normalized to weight of spleen (right panel) from *LysMCre* (n=4; mean ± s.e.m.) and *LysMCre;Arnt*$^{fl/fl}$ (n=4; mean ± s.e.m.) mice without DSS challenge. Two-way ANOVA, *p<0.05, **p<0.01, ***p<0.001, and ****p<0.0001.

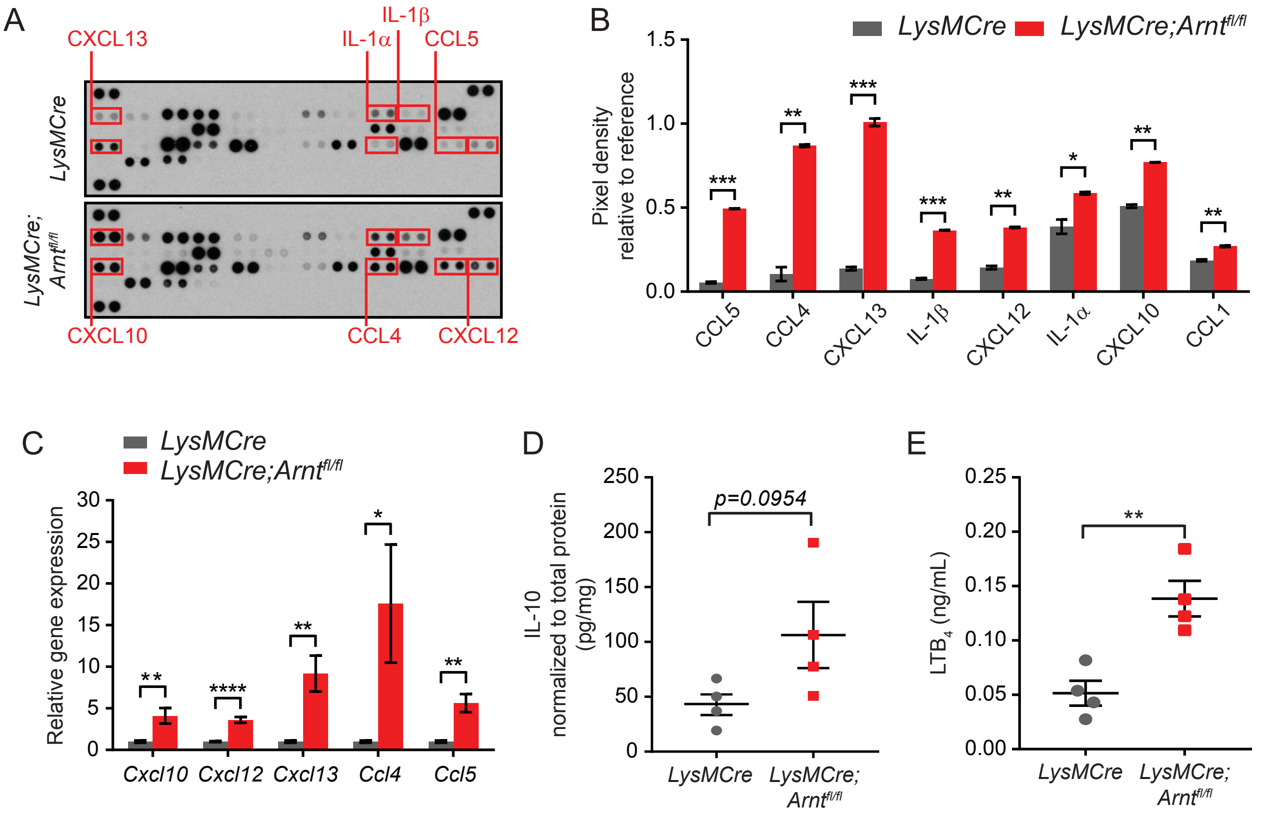

Supplementary Figure S2. Myeloid HIF-α/ARNT heterodimer deficiency promotes secretion of pro-inflammatory cytokines and LTB_4 in the colon. **(A)** Cytokine array using pooled colonic explant supernatants from DSS-treated *LysMCre* and *LysMCre;Arnt$^{fl/fl}$* mice sacrificed on Day 8. **(B)** Quantification of cytokine array based on density. **(C)** RT-qPCR analysis of genes encoding several pro-inflammatory cytokines showed upregulation in **(B)**, N=6 for each genotype; mean ± s.e.m. (**D**) ELISA analysis of IL-10 from Day 8 colonic explant supernatants (n=4 for each genotype; mean ± s.e.m.) (**E**) Liquid chromatography–mass spectrometry analysis of LTB_4 in *LysMCre* and *LysMCre;Arnt$^{fl/fl}$* (n=4 for each genotype, mean ± s.e.m.) colonic explant supernatants from Day 8. Student's t-test, *p<0.05, **p<0.01, ***p<0.001, and ****p<0.0001.

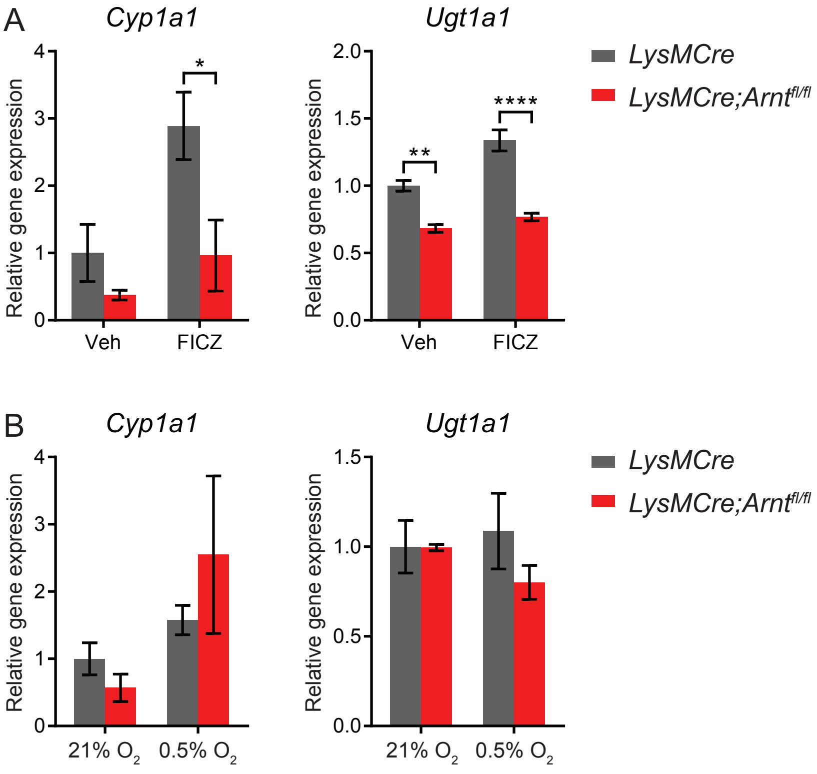

Supplementary Figure S3. ARNT deficiency suppresses AhR ligand-induced AhR target gene expression, but does not affect their expression under hypoxia. **(A-B)** RT-qPCR analysis of *Cyp1a1* and *Ugt1a1* in BMDMs treated with FICZ (100 nM) under normoxia **(A)** and BMDMs cultured under normoxia or hypoxia **(B)** for 24 hours (n=3 for each genotype under each condition; mean ± s.e.m.) Two-way ANOVA; *p<0.05, **p<0.01, ***p<0.001, and ****p<0.0001.

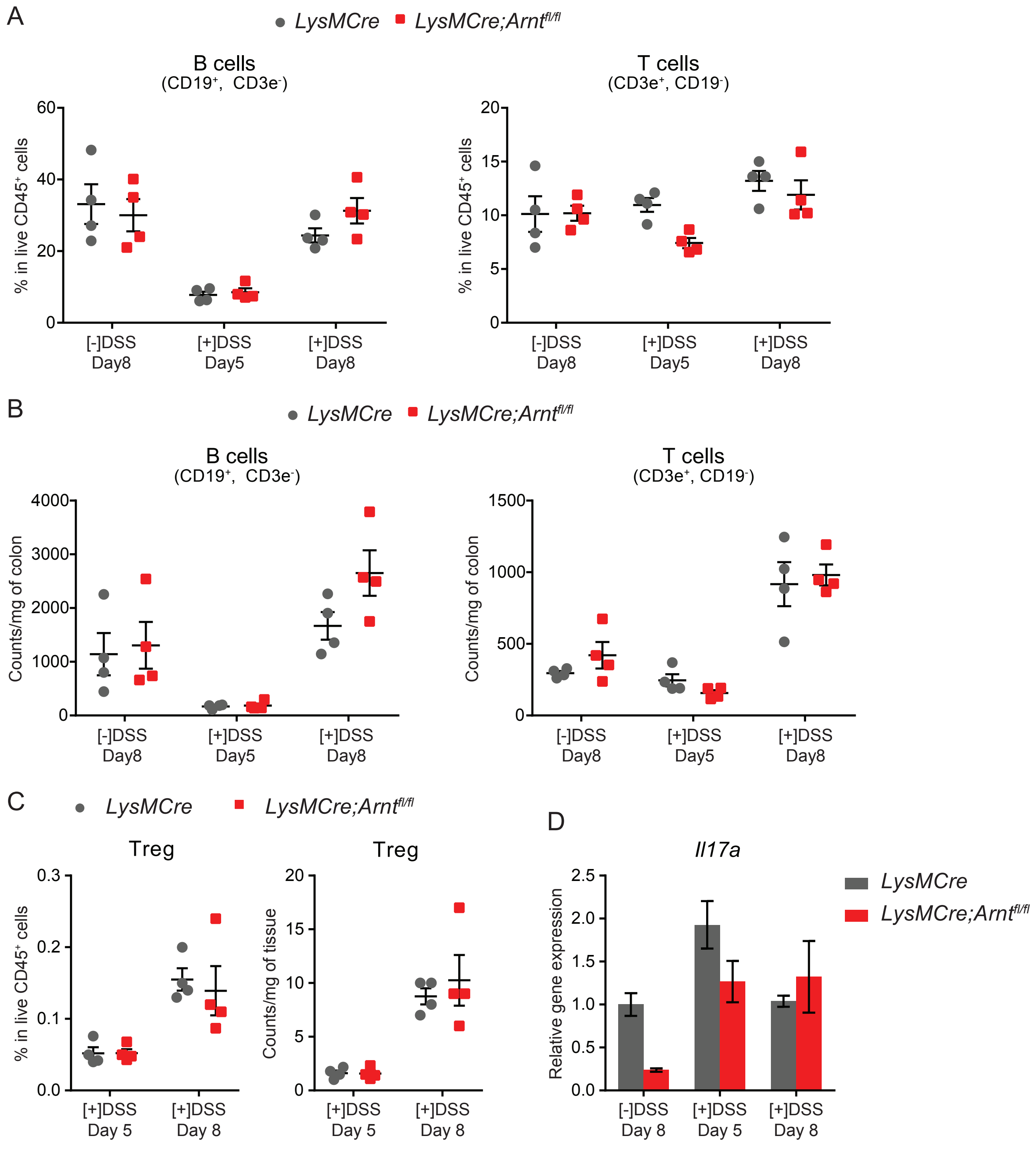

Supplementary Figure S4. Myeloid HIF deficiency does not alter composition of T cells and B cells in lamina propria. **(A-B)** Percentage in $CD45^+$ cells **(A)** and absolute cell counts normalized to colon weight **(B)** of B cells and T cells from *LysMCre* and *LysMCre;Arnt$^{fl/fl}$* mice. **(C)** Percentage in $CD45^+$ cells and absolute cell counts normalized to colon weight of Tregs from *LysMCre* and *LysMCre;Arnt$^{fl/fl}$* mice. (**D**) RT-qPCR analysis of *Il17a* in colonic tissues from *LysMCre* and *LysMCre;Arnt$^{fl/fl}$* mice. Data presented as mean ± s.e.m. Two-way ANOVA, *p<0.05, **p<0.01, ***p<0.001, and ****p<0.0001.

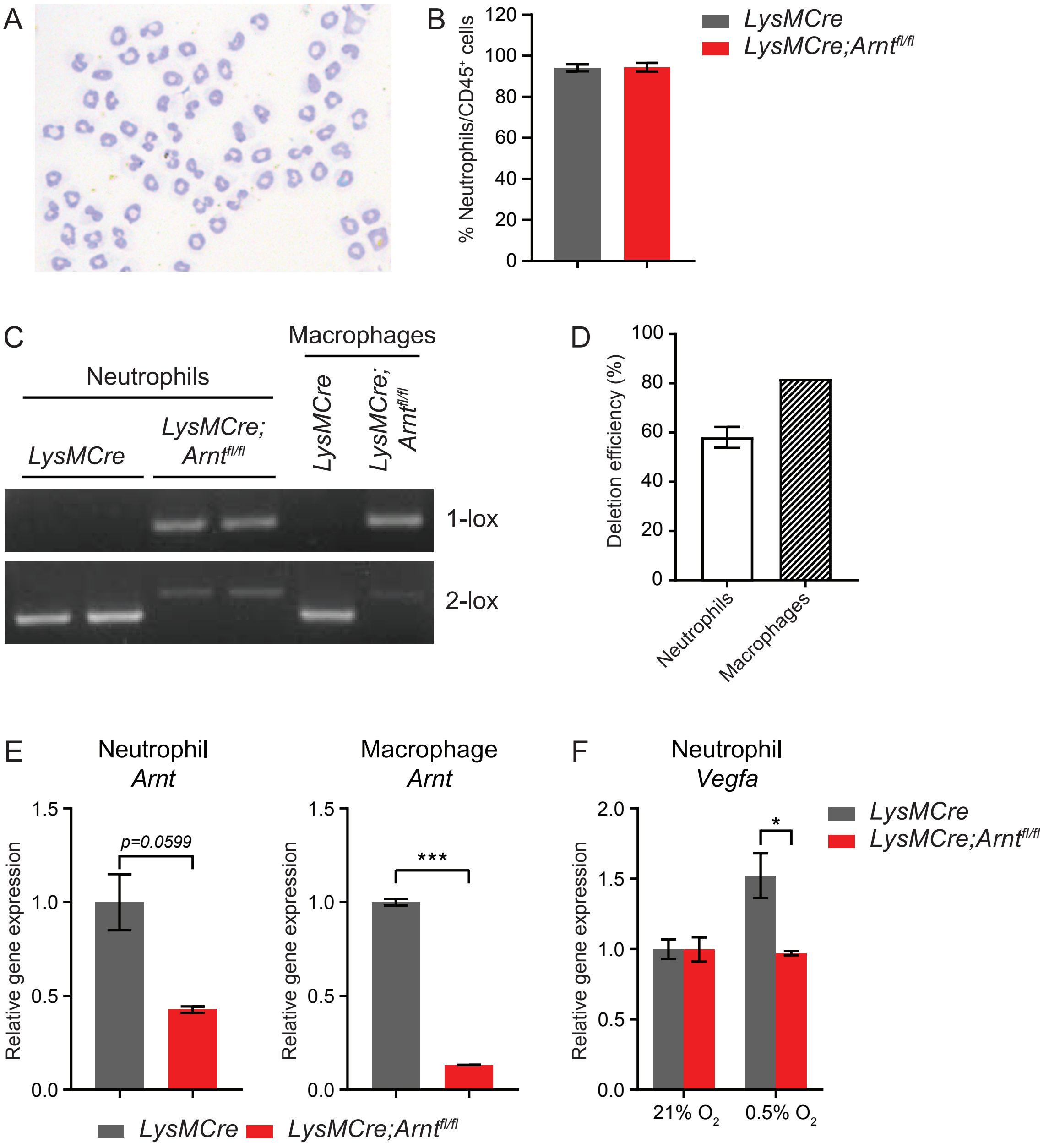

Supplementary Figure S5. *Arnt* is partially deleted in neutrophils. **(A)** Example of neutrophils from bone marrow after enrichment. **(B)** Flow cytometry confirmation of high neutrophil percentage after enrichment from bone marrow of *LysMCre* and *LysMCre;Arnt$^{fl/fl}$* mice. **(C)** PCR analysis of genomic DNA prepared from BMDNs and BMDMs of *LysMCre* and *LysMCre;Arnt$^{fl/fl}$* mice. The Cre recombinase-mediated recombination of the conditional allele (2 *lox*) leaves behind a single LoxP (1 *lox*) site. **(D)** Quantification of deletion efficiency based on **(C)** using density. Deletion efficiency = (1 *lox* density)/(1 *lox* density + 2 *lox* density). **(E)** RT-qPCR analysis of *Arnt* in BMDNs (left panel) (n=3 for each genotype; mean ± s.e.m.) and BMDMs (right panel) (n=3 for each genotype; mean ± s.e.m.) Student's t-test, ***p<0.001 **(F)** RT-qPCR analysis of *Vegfa* in BMDNs cultured under normoxia or hypoxia for 24 hours (n=3 for each genotype; mean ± s.e.m.). Two-way ANOVA, *p<0.05.

Lin et al. Supplementary Figure S6

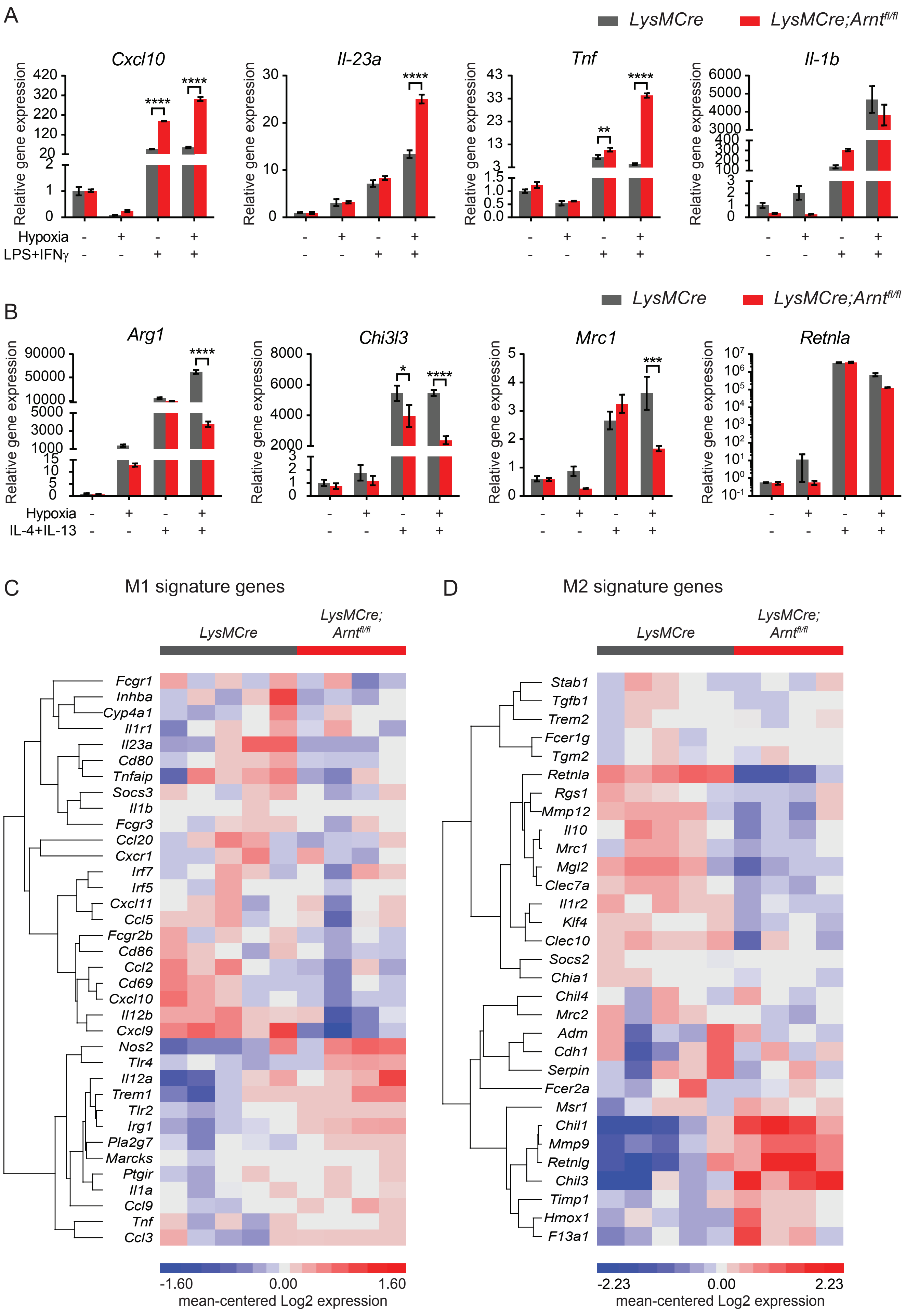

Supplementary Figure S6. Myeloid HIF deficiency favors M1 polarization of macrophages *in vitro*, but not *in vivo*. **(A-B)** RT-qPCR analysis of M1 **(A)** and M2 **(B)** signature genes in BMDMs (n=3 for each genotype, mean ± s.e.m.) cultured under hypoxia with either M1 stimuli (5 ng/mL LPS+1 ng/mL IFNγ) or M2 (5 ng/mL IL-4+5 ng/mL IL-13) for 24 hours. Two-way ANOVA; $^*p<0.05$, $^{**}p<0.01$, $^{***}p<0.001$, and $^{****}p<0.0001$. **(C-D)** Heatmap of M1 **(C)** and M2 **(D)** signature gene expression based on microarray analysis of sorted lamina propria macrophages from *LysMCre* and *LysMCre;Arnt*$^{fl/fl}$ mice. These mice were treated with DSS for 5 days and sacrificed on Day 8.

KEGG gene set	p-value	FDR q-value
Notch signaling pathway	7.23 e-5	1.06 e-2
Arachidonic acid metabolism	1.36 e-4	1.06 e-2
Retinol metabolism	1.82 e-4	1.06 e-2

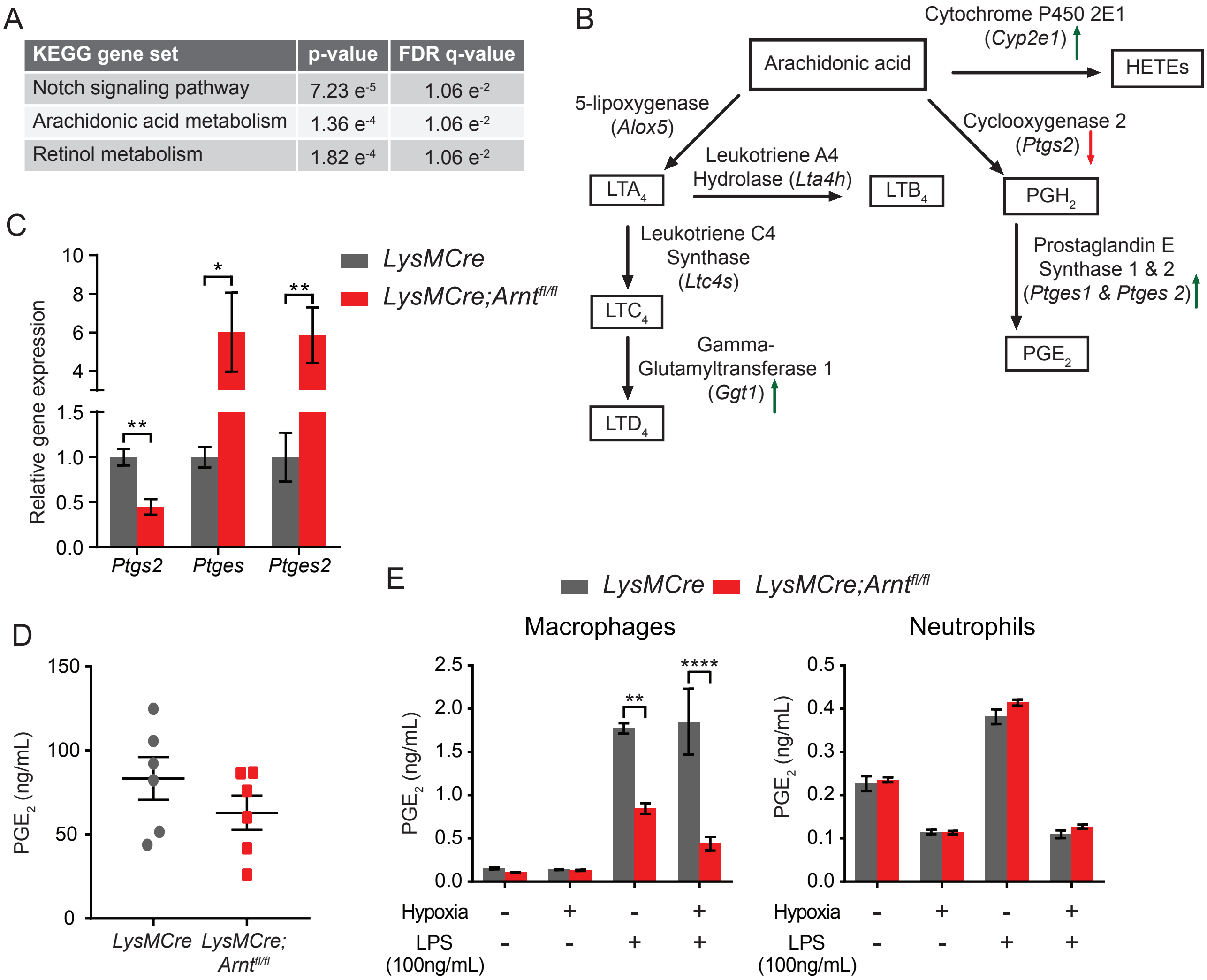

Supplementary Figure S7. Myeloid HIF deficiency alters PGE_2 production in macrophages and in the colon. **(A)** KEGG pathway analysis of significantly upregulated genes in *LysMCre;Arnt*$^{fl/fl}$ lamina propria macrophages. **(B)** Simplified schematic of arachidonic acid metabolism. Green upward arrow indicates upregulation of gene expression, and red downward arrow indicates downregulation. **(C)** RT-qPCR analysis of *Ptgs2*, *Ptges1*, and *Ptges2* expression in *LysMCre* and *LysMCre;Arnt*$^{fl/fl}$ lamina propria macrophages. Student's t-test, *p<0.05, and **p<0.01 **(D-E)** Liquid chromatography–mass spectrometry analysis of PGE_2 in **(D)** *LysMCre* and *LysMCre;Arnt*$^{fl/fl}$ colonic explant supernatants from Day 8 and in **(E)** supernatants collected from BMDMs (left, n=3 for each genotype, mean ± s.e.m.) and BMDNs (right, n=3 for each genotype, mean ± s.e.m.) cultured under normoxia or hypoxia, with or without LPS stimuli (100 ng/mL) for 24 hours. Two-way ANOVA; **p<0.01, and ****p<0.0001.
